# Effect of Universal Credit on young children’s mental health: quasi-experimental evidence from Understanding Society

**DOI:** 10.1136/jech-2024-222293

**Published:** 2024-08-17

**Authors:** Huihui Song, Anwen Zhang, Benjamin Barr, Sophie Wickham

**Affiliations:** 1 Department of Public Health, Policy and Systems, University of Liverpool, Liverpool, UK; 2 Adam Smith Business School, University of Glasgow, Glasgow, UK

**Keywords:** HEALTH POLICY, ECONOMICS, CHILD HEALTH, MENTAL HEALTH

## Abstract

**Background:**

Child mental health has become an increasingly important issue in the UK, especially in the context of significant welfare reforms. Universal Credit (UC) has introduced substantial changes to the UK’s social security system, significantly impacting low-income families. Our aim was to assess the effects of UC’s introduction on children’s mental health for families eligible for UC versus a comparable non-eligible sample.

**Methods:**

Using Understanding Society data from 5806 observations of 4582 children (aged 5 or 8 years) in Great Britain between 2012 and 2018, we created two groups: children whose parents were eligible for UC (intervention group) and children whose parents were ineligible for UC (comparison group). Child mental health was assessed using a parent-reported Strengths and Difficulties Questionnaire. The OR and percentage point change in the prevalence of children experiencing mental health difficulties between the intervention group and the comparison group following the introduction of UC were analysed. We also investigated whether the utilisation of childcare services and changes in household income were mechanisms by which UC impacted children’s mental health.

**Results:**

Logistic regression results demonstrated that the prevalence of mental health problems among eligible children whose parents were unemployed increased by an OR of 2.18 (95% CI 1.14 to 4.18), equivalent to an 8-percentage point increase (95% CI 1 to 14 percentage points) following the introduction of UC, relative to the comparison group. Exploring potential mechanisms, we found neither reduced household income nor increased use of childcare services, which served as a proxy for reduced time spent with parents, significantly influenced children’s mental health.

**Conclusions:**

UC has led to an increase in mental health problems among recipient children, particularly for children in larger families and those aged 8. Policymakers should carefully evaluate the potential health consequences for specific demographics when introducing new welfare policies.

**WHAT IS ALREADY KNOWN ON THIS TOPIC**

- Previous research has focused on the health implications of Universal Credit for adult populations; however, a gap remains regarding evaluations of the policy’s influence on the mental health of children post implementation.

**WHAT THIS STUDY ADDS**

- Employing a quasi-experimental study design, this research identified that the implementation of UC was linked to an increase in children’s mental health issues. These findings suggested that benefits policy shocks affected not only adult recipients but also extended to their children, highlighting the broader impact of such policy changes on family well-being.

**HOW THIS STUDY MIGHT AFFECT RESEARCH, PRACTICE OR POLICY**

- In light of the evidence demonstrating the adverse effects of welfare changes on children’s mental health, it is imperative to establish a comprehensive health impact assessment of children’s well-being within any welfare reform evaluation. Furthermore, the more pronounced impact of UC on children in larger families and those aged 8 years underscores the importance of considering household-specific effects in policy implementation. The health outcomes of children should be a central consideration when redesigning welfare systems.

## Introduction

 Childhood is a critical phase for mental health, characterised by rapid brain growth and development.^1^ During this period, children acquire cognitive skills that shape their future mental well-being and are essential for assuming adult roles in society. This underscores the vital importance of providing children with the best possible start in life, particularly in early childhood. In the UK, there has been a concerning trend of worsening mental health among young children. For example, rates of mental health issues among children aged 5–10 years rose from 9% in 2017 to 14% in 2020.^2^ Addressing the causes of this increase is a public health priority.

**Figure 1:**
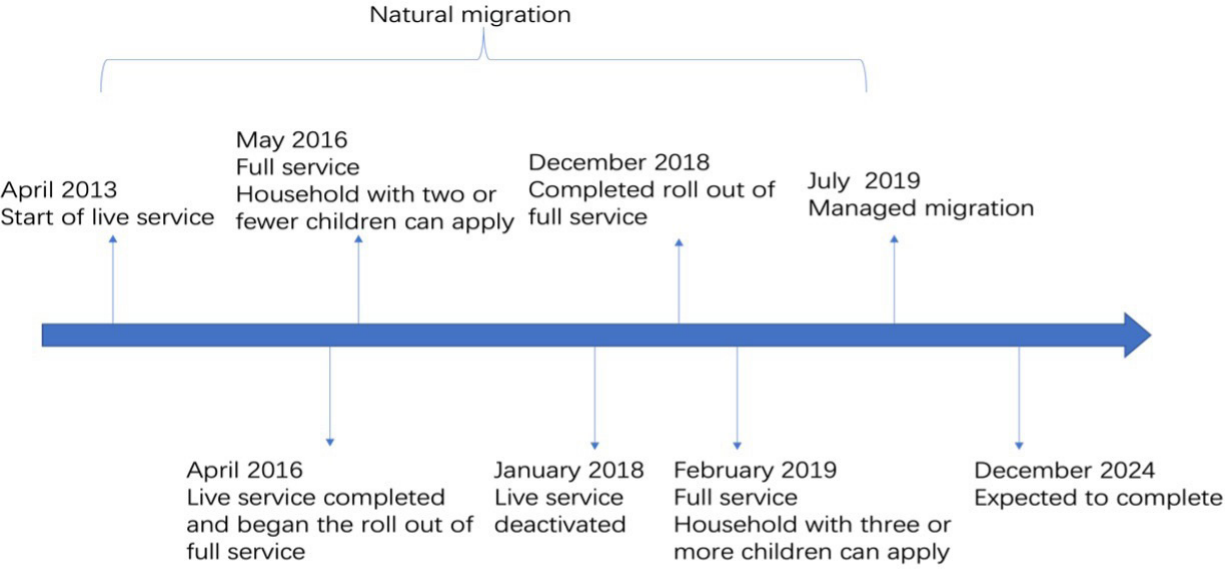
Timeline of UC. Note: Live service started in April 2013 in the North West. It did not involve online applications; only single, childless, unemployed adults without housing costs were eligible initially. In April 2016, full service commenced, accepting new claims from all types of claimants and concluded in December 2018. Natural migration refers to situations when existing claimants of legacy benefits and tax credits experienced a change in circumstances, such as unemployment, and were migrated to UC. Managed migration refers to the process of transferring the remaining claimants of legacy benefits and tax credits to UC. Source: National Audit Office (2018).^4^ UC, Universal Credit.

Policy actions can yield unintended consequences, and welfare reform stands out as a potential contributor to such mental health outcomes.^3^ Universal Credit (UC) is arguably the biggest overhaul of the welfare system in the UK since the Beveridge reforms of the 1940s. UC has been gradually implemented across the UK for different groups of people. The rollout of UC commenced in April 2013, with eligibility extended to families with children starting in May 2016 (figure 1 illustrates the timeline of the national expansion of the UC rollout). The Department for Work and Pensions data show that as of February 2022, there were 3.8 million children in over 2 million households who were receiving UC, accounting for 49% of all households under UC. Three-quarters of families with children on UC had a child of primary school age or younger.^4^

UC has been criticised for its digitalised implementation style, wait for first payment and increased use of conditionality and sanctions. Studies have found negative impacts of UC on employment outcomes,^5^ debt,^6^ food bank usage,^7^ housing insecurity^8^ and higher crime rates.^9 10^ There are several papers, both quantitative and qualitative, that have explored the impact of UC on the mental health and psychological distress of working-age adults, finding that individuals entering UC experienced a deterioration in their mental health.^11^,^13^ There have not, however, to our knowledge, been any previous studies investigating the impact on children. Therefore, understanding the impact of UC on child mental health is now urgently needed, given the rise in child mental illness we are seeing in the UK.^2^

**Figure 2:**
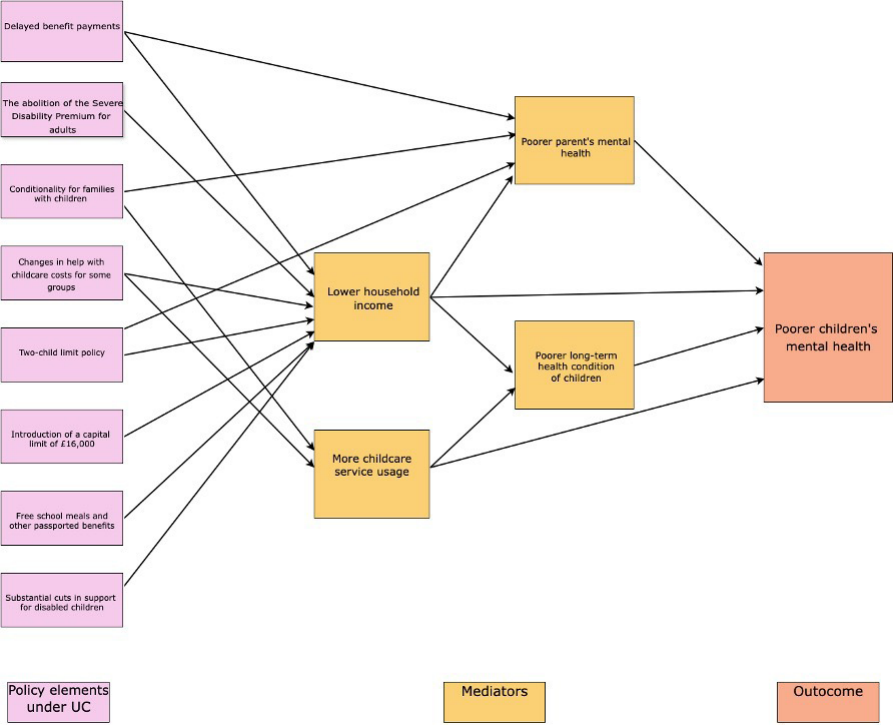
Possible pathways to poor mental health outcomes of young children under UC. Note: This simplified figure illustrates potential pathways leading to adverse mental health outcomes in young children under UC. For a more detailed illustration of these pathways, please refer to online supplemental appendix 1. UC, Universal Credit.

In exploring the mechanisms by which UC impacts children’s mental health, there are multiple factors to consider (see figure 2). For example, a reduction in household income under UC may be detrimental to children’s well-being and development.^14^ Compared with entitlements under the tax credit system, the majority of working families are worse off under UC, experiencing an average loss of entitlement of £41 per week.^15^ In addition, under UC, the introduction of the two-child limit restricts child-related benefits to the first two children in a family with a third or additional baby since April 2017. This means that families lose roughly £237 per child, which reduces the overall income available to households with larger families and has pushed more families into poverty or deeper into poverty. Moreover, the requirement for compulsory intensive job searches for unemployed or low-income claimants may lead parents to rely more on childcare services, reducing the time spent with their children, which could affect children’s mental health.

Hence, there is an urgent need to understand both the potential differential effects of UC for different groups of children and the potential mechanisms through which UC impacts children’s mental health to inform future policy implementation. Several important pathways through which UC may impact children’s mental health are depicted in figure 2.

In this paper, we examined the potential impact of UC on young children’s socioemotional behavioural difficulties, which have been recognised as a critical factor in understanding mental health outcomes,^16^ with a growing body of research consistently identifying it as a pivotal marker on the pathway to mental illness.^17^ We compared the socioemotional behavioural difficulties of children whose parents were unemployed and therefore eligible for UC to those not eligible for UC before and after UC became available for families with children in 2016. We also explored if there were differential effects of the introduction of UC for younger versus older children and single-child versus multiple-child households. Finally, we explored whether the reduction in household income or changes in childcare service usage were the pathways through which UC impacted children’s socioemotional behavioural difficulties.

## Methods

### Study design and participants

We used data from the UK Household Longitudinal Study (UKHLS). The UKHLS is a large and nationally representative panel survey of approximately 40 000 households. It includes information on households’ social, economic and demographic status, health, employment, and social benefits across the UK from 2009 onwards.^18^ The Strengths and Difficulties Questionnaire (SDQ) has been collected in the UKHLS since Wave 3 and is only asked of children aged 5 and 8 years. Therefore, we included data from 2012 to 2018, covering eight waves of data. According to the inclusion and exclusion criteria, data from 5806 observations of 4582 children aged 5 and 8 years were included in the study population. A flowchart of participants and details of the study sample can be found in online supplemental appendix 2.

### Eligibility and policy exposure

From May 2016, households with children became eligible for UC in England, Scotland and Wales.^19^ We took a conservative approach to eligibility and classified children’s exposure to UC based on their parents' unemployment status. Children were assigned to the intervention group if at least one of their working-age parents (18–64 years) identified as unemployed and therefore eligible to receive UC. They were assigned to the comparison group in a given wave if their parents identified as anything other than unemployed. Eligibility could vary over time. The interview year was used to determine the period before (<2016) and after policy exposure (≥2016).

### Outcomes

The primary outcome of interest was young children’s socioemotional behavioural difficulties using the parent-reported SDQ. This is a short behavioural screening questionnaire for children and was only asked of parents whose young children were aged 5 and 8 years. The composition of the SDQ is detailed in online supplemental appendix 3. A total difficulties score was created by summing the first four subscales (range 0–40). We used a dichotomised score and constructed a dummy variable indicating mental difficulty, where 0–16 indicated no difficulties and 17–40 indicated socioemotional behavioural difficulties.^20^ The dichotomised score better reflects a clinically meaningful effect on child’s mental health, and it is likely that the effect of the policy on social and behavioural difficulties is non-linear, potentially having a greater effect at higher levels of SDQ score. We tested this assumption using quantile regression (see online supplemental appendix 4) and repeated the analysis using the continuous score (see online supplemental appendix 7).

### Covariates

Following the literature,^21^ continuous covariates included the logarithm of household inflation‐adjusted income (household income was measured as the logarithm of the contemporaneous monthly net income from the labour market and all other sources taking away any taxes, deductions and benefits in GB 2010 prices) and the mother’s mental health (measured using the 12‐item General Health Questionnaire).^22^

Categorical covariates included the child’s age (either 5 or 8), gender (female=0 and male=1), long-term health condition (‘Excellent’ compared with ‘very good’, ‘good’, ‘fair’ and ‘poor’), the mother’s education level (‘Degree’ compared with ‘other higher’, ‘A levels’, ‘GCSE’ and ‘no/other qualification’), and whether there was only one child in the family (only one child in family=1 and additional children in family=0). Childcare utilisation was measured based on maternal reports, with a value of 1 indicating that the mother reported using childcare services, and 0 otherwise.

### Statistical analysis

#### Main analysis

To understand if the introduction of UC has had an effect on child socioemotional behavioural difficulties of parents eligible for UC, we first analysed whether the trends in socioemotional behavioural difficulties ran in parallel prior to the intervention. This comparison of trends in the outcome focused on the percentage of children with mental health issues, specifically those with SDQ scores equal to or exceeding 17. This comparison was conducted between the intervention and comparison groups during the preintervention period.

Next, we employed a generalised difference-in-differences framework and logistic models to identify the treatment effect of UC on children’s socioemotional behavioural difficulties between 2012 and 2018. This analysis compared children of parents eligible for UC with those of ineligible parents, adjusting for the covariates described above. Therefore, changes in mental health for the children limit potential biases after controlling for covariates.

We conducted several robustness tests to investigate whether our results were sensitive to model specifications. First, we repeated our main analysis using an alternative approach to eligibility. We classified children’s exposure based on their parent’s reports of working-age benefits. Children were assigned to the intervention group if at least one of their parents reported receiving either UC or one of the six legacy benefits and were therefore eligible to receive (or move onto) UC. They were assigned to the comparison group in a given wave if their parents did not report receiving UC or legacy benefits.

Second, we repeated the main analysis using the continuous measure of SDQ as the outcome. For this model, we used a linear rather than logistic regression model. Third, we constructed a ‘stable treatment’ status for children in order to implement canonical difference-in-differences to overcome potential issues around treatment status staggering. Children were assigned to the intervention group if their parents were unemployed for any period. Once assigned, they were considered to belong to the intervention group for the entire period. The comparison group was defined as children whose parents were always employed, which allowed us to construct a time-invariant comparison group. Fourth, we repeated the analysis, excluding families with more than two children, as UC initially only allowed families with two or fewer children to apply. Fifth, we repeated our main analysis and excluded the top 25% of households with the highest income to improve comparability between the intervention and comparison groups. Sixth, we only included children with two or more observations of the outcome in a linear probability model with individual fixed effects to re-estimate the main findings. Seventh, we used propensity score matching with bootstrapping to overcome demographic variation between the intervention and comparison groups. Eighth, we used the linear ramp model to explore the potential temporal variation in the rollout of UC. Finally, to overcome potential bias in the missing data (ie, structural missingness in the outcome variable and other forms of non-random missingness), we have repeated the main analysis using multiple imputation and inverse probability weighting (IPW).

#### Exploring heterogeneity effects and mechanisms

We conducted two heterogeneity tests to explore if there were differential effects based on child’s age and household composition, specifically whether the household had only one child or multiple children. We repeated the main analysis using subgroups to explore variability in effects.

To explore the mechanism through which UC potentially affected children’s socioemotional behavioural difficulties, we investigated two policy elements embedded in UC. First, we used the utilisation of childcare as a potential proxy for reduced time spent with parents (and potentially the changes in conditionality under UC). Second, we explored changes in household income as a proxy for changes in benefit income. We repeated the main analysis, substituting the socioemotional behavioural outcome with the mechanism variable. A detailed description of all methodologies is described in online supplemental appendix 4.

## Results

We included 5806 observations from 4582 children (aged 5 or 8 years) in England, Wales and Scotland who participated in the UKHLS between 2012 and 2018. The baseline characteristics of the intervention and comparison groups in the years prior to UC’s introduction are presented in table 1 (online supplemental appendix 6 outlines the number of observations in the intervention and comparison groups). The socioemotional behavioural difficulty was more prevalent in the intervention group compared with the comparison group. Consistently, the average SDQ scores for the intervention group were 1.8 points higher than those of the comparison group. The comparison group used childcare services more frequently and had a higher household income. There were no large differences between participants in the intervention group and the comparison group in terms of age and gender. Children in the intervention group exhibited worse long-term health conditions and lived in households with more than one child. Additionally, the intervention group had a higher prevalence of mothers experiencing mental health issues and lower levels of educational attainment.

**Table 1:** Baseline characteristics in the years before Universal Credit was introduced

	Comparison group	Intervention group	P value
	(N=3396)	(N=222)	
SDQ			
Socioemotional behavioural difficulties	256 (7.5%)	33 (14.9%)	–
No difficulties	3140 (92.5%)	189 (85.1%)	0.0029
continuous SDQ score	7.8 (0.1)	9.6 (0.4)	0.0001
Childcare usage			
Yes	1662 (48.9%)	42 (18.9%)	–
No	1734 (51.1%)	180 (81.1%)	<0.0001
Log of household income	8.2 (0.1)	7.6 (0.1)	<0.0001
Age, years			
Age 5	1758 (51.8%)	107 (48.2%)	–
Age 8	1638 (48.2%)	115 (51.8%)	0.3046
Gender			
Male	1755 (51.7%)	108 (48.6%)	–
Female	1641 (48.3%)	114 (51.4%)	0.3834
Long-term health condition			
Excellent	2003 (59.0%)	118 (53.2%)	–
Very good	1031 (30.4%)	67 (30.2%)	–
Good	276 (8.1%)	26 (11.7%)	–
Fair	68 (2.0%)	9 (4.1%)	–
Poor	18 (0.5%)	2 (0.9%)	0.0182
Mothers’ degree			
Degree	1256 (37.0%)	40 (18.0%)	–
Other higher	514 (15.1%)	19 (8.6%)	–
A level	686 (20.2%)	29 (13.1%)	–
GCSE (General Certificate of Secondary Education)	723 (21.3%)	82 (36.9%)	–
Other qualification	119 (3.5%)	15 (6.8%)	–
No qualification	98 (2.9%)	37 (16.7%)	<0.0001
Mothers’ mental health (General Health Questionnaire-12 score)	1.7 (0.1)	2.83 (0.3)	<0.0001
Single child in the household			
Yes	455 (13.4%)	17 (7.7%)	–
No	2941 (86.6%)	205 (92.3%)	0.0025

Note: Data are n (%), or mean (SD). We defined clinically significant psychological distress as a score of greater than 16 on the SDQ.

SDQStrengths and Difficulties Questionnaire

**Figure 3:**
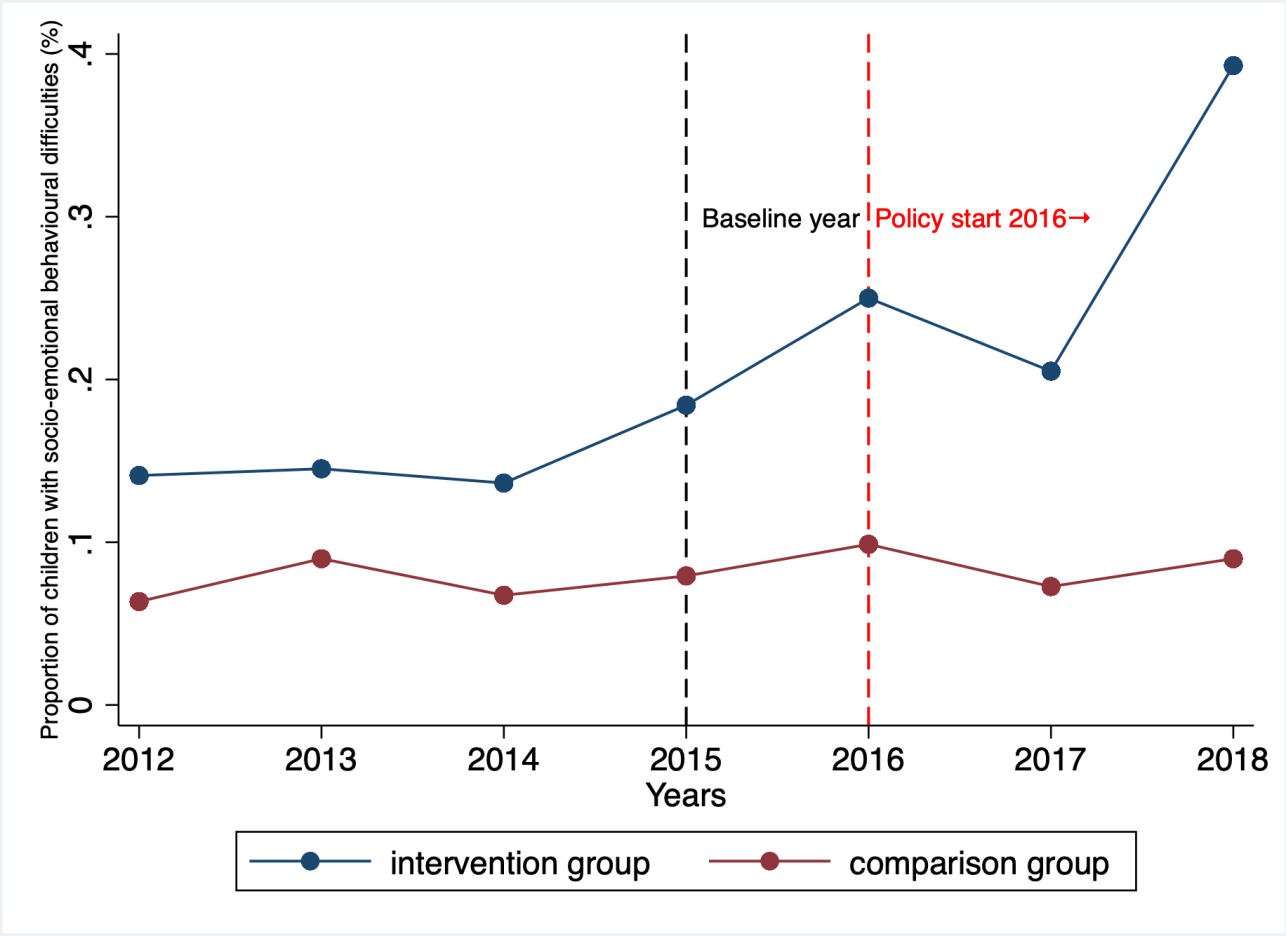
Graphical representation of socioemotional behavioural difficulties in the intervention and comparison groups before and after Universal Credit was introduced. (Observing parallel trends in the preintervention period.)

The trend in the proportion of children with socioemotional behavioural difficulties in both the intervention and comparison groups before and after the introduction of UC is displayed in figure 3. While the intervention and comparison groups differed in terms of their difficulties prior to UC, this difference, however, should not introduce bias in the analysis, as the difference between the two groups would persist at the same level in the absence of UC (see online supplemental appendix 5 for full regression results of the parallel trend analysis). The parallel trend graph suggested that a greater number of children in the intervention group experienced mental health issues compared with the comparison group following the implementation of UC.

The difference-in-difference results in table 2 indicated that UC exacerbated children’s socioemotional behavioural difficulties in households with unemployed parents. The effect of UC was to increase the prevalence of difficulties by an OR of 2.18 (95% CI 1.14 to 4.18), equivalent to an 8-percentage point increase (95% CI 1 to 14) among eligible children.

**Table 2:** Difference-in-difference estimates of the impact of UC on children’s socioemotional behavioural difficulties

	Treatment effect	Estimate (95% CI)	P value
Using unemployment to define eligibility	OR, change in odds of difficulties associated with the intervention (SDQ caseness)	2.18 (1.14 to 4.18)	0.02
	Percentage point change in prevalence of difficulties	0.08 (0.01 to 0.14)	0.02

Note: Data are estimates of the change in outcomes among children eligible for Universal Credit after its introduction relative to the change in outcomes among those not affected by the policy change. The model based on equations is presented in online supplemental appendix 4Appendix 4, and full model results are included in the online supplemental appendix 6Appendix 6.

SDQStrengths and Difficulties Questionnaire

**Figure 4:**
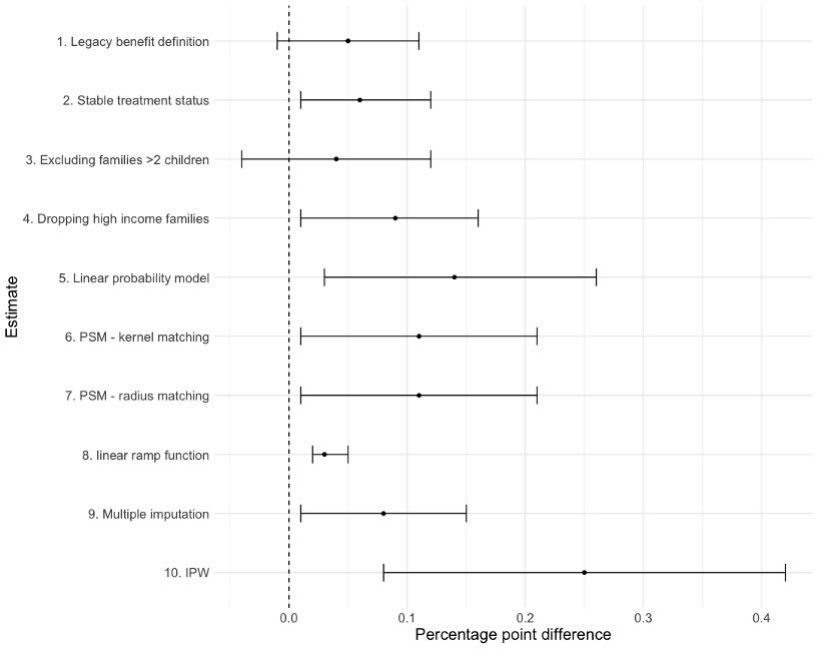
Outcomes of a series of robustness analyses with 95% CIs. Note: When employing continuous SDQ scores to assess children’s mental health conditions, the changes were measured on a different scale rather than in percentage points; thus, this outcome was not included in the graph. However, the results were similar, showing a 1.40 SDQ score increase (95% CI 0.49 to 2.39) for the intervention group after the introduction of Universal Credit. The results are presented in online supplemental appendix 7. SDQ, Strengths and Difficulties Questionnaire.

The outcomes of a series of robustness analyses are presented in figure 4. These included the alternative definition of eligibility criteria, adjustments to the scope of the study population, and the utilisation of different model specifications. Except for the analysis limited to families with fewer than two children, which suggested a weaker effect, the results remained consistent across various checks. More detailed results, including parallel trends and regression outcomes, are provided in online supplemental appendix 7.

The results of the heterogeneity effects showed that following the implementation of UC, the prevalence of socioemotional behavioural difficulties increased by an OR of 2.40 (95% CI 1.20 to 4.83), equivalent to a 9-percentage point increase (95% CI 2 to 16 percentage points) for eligible children in families with two or more children while exhibiting an insignificant effect on eligible children in one-child families (table 3). Additionally, the results suggested that UC negatively impacted children aged 8 (95% CI 5 to 26 percentage points).

**Table 3:** Heterogeneity results of the impact of Universal Credit on children’s mental health

	Estimate (95% CI)	P value	Estimate (95% CI)	P value
	Children aged 5 years (N=2929)		Children aged 8 years (N=2876)	
OR, change in odds of psychological distress associated with the intervention	0.99 (0.36 to 2.72)	0.99	4.15 (1.69 to 10.17)	0.00
Percentage point change in prevalence of psychological distress	−0.00 (-0.08 to 0.08)	0.98	0.16 (0.05 to 0.26)	0.00
	One-child families (n=745)		Families with two or more children (n=5061)	
OR, change in odds of psychological distress associated with the intervention	1.12 (0.16 to 7.90)	0.91	2.40 (1.20 to 4.83)	0.01
Percentage point change in prevalence of psychological distress	0.01 (-0.15 to 0.16)	0.93	0.09 (0.02 to 0.16)	0.02

Note: online supplemental appendix 8Appendix 8 provides the full results and parallel trends for the heterogeneity test.

Our exploration of the mechanism through which UC potentially affected children’s socioemotional behavioural difficulties found that neither the use of childcare services nor a reduction in household income were the main contributors to children’s experiences of worse socioemotional behavioural difficulties (see online supplemental appendix 9).

## Discussion

This paper has demonstrated that the implementation of UC has exacerbated socioemotional behavioural difficulties in children. This corresponded to an 8-percentage point (95% CI 1 to 14 percentage points) increase in the proportion of children with parents eligible for UC based on their employment status experiencing socioemotional behavioural difficulties. This estimation served as a conservative estimate, as some individuals in the comparison group might also have been eligible to apply for UC for reasons other than unemployment, although accounting for only 2% of the comparison group.^11^ The findings were strengthened by the robustness tests showing similar effects from different model specifications.

This study emphasised that children in larger families and those aged 8 years may be more susceptible to the impacts of welfare reform, underscoring the importance of intervention strategies. To understand why children were affected by UC, we analysed the treatment effects on two mediators linked to the subelements of UC. The results suggested that neither lower household income nor parents’ use of childcare services were the main factors that caused the observed deteriorating child mental health. This might be because the income measure, based on survey-reported data, does not fully capture the effect, especially at very low-income levels where negative impacts might be concentrated. Additionally, delays in benefit payments, sanctions under UC and the anticipation of moving into employment could be alternative pathways affecting children’s mental health.

Our study adds to the growing body of evidence of the adverse effects of UC on various socioeconomic aspects^5^,^10^ that have focused on the experiences of adults. Regarding the mental health of adults, both quantitative and qualitative studies have explored the impact of UC on working-age adults, consistently finding a decline in mental health among individuals transitioning to UC.^11^,^13^ However, there is a gap in research concerning the impact of UC on children’s mental health.

Our research endeavours to augment the existing body of knowledge by furnishing longitudinal evidence that illuminates the mental health ramifications associated with the transition to UC for children with unemployed parents. By doing so, our study underscores that the unintended consequences of UC extend beyond the recipients themselves, also impacting the mental well-being of children.

This study has some limitations. First, the intervention group had a small sample size, introducing potential uncertainty. The CI suggested that the true impact of UC on children’s mental health may range between 1 and 14 percentage points. Second, the implementation of UC in the UK follows a staggered full-service rollout schedule, leading to variations in the timing of application for eligible children. However, estimating the impact of the UC rollout on children’s mental health is constrained by the limitation of a small sample size within a singular district. Third, we used reported unemployment. However, not all unemployed individuals received UC, and some participants in the comparison group may have become eligible over the course of the analysis, although only a small proportion (<2%) of the comparison group was affected.^11^ Alternative definitions, however, indicated similar results. Fourth, the prevalence of missing data and attrition posed common challenges in longitudinal datasets and natural policy methodologies. Lastly, our measure of use of childcare services may not have been an accurate measure of time spent in childcare services and did not reflect the quality of those arrangements, thus hindering our ability to determine the potential mechanisms involved.

Considering the adverse influence of UC on children’s mental health, as outlined in this paper, it is imperative for future government policies in the UK and other countries to consider the well-being of children when reforming the welfare system. The mechanisms for this effect remain unclear. Further research should aim to understand the experience of families with children using UC and the potential pathways for negative and positive effects on child well-being, adapting the service to maximise positive benefits. Furthermore, specific policies related to children, including parental conditionality and the benefit cap, require further research to explore their impact on children and young people. Policymakers should give greater consideration to the health impact of changes to welfare systems on children.

## supplementary material

10.1136/jech-2024-222293 online supplemental appendix 1

## Data Availability

Data are available upon reasonable request.
