# Supplementary material for: Effect of Universal Credit on young children’s mental health: quasi-experimental evidence from Understanding Society
Source: J Epidemiol Community Health. 2024 Aug 17;78(12):e222293. doi: 10.1136/jech-2024-222293 (PMC11672006; doi:10.1136/jech-2024-222293)
Supplement: online supplemental appendix 1 [file jech-78-12-s001.pdf]

The effect of Universal Credit on young children's mental health: Quasi-experimental evidence from Understanding Society

Huihui Song, Anwen Zhang, Ben Barr, Sophie Wickham.

List of Appendix

Appendix 1. How does Universal Credit affect children's mental health? 2

Appendix 2 Flowchart of the child population and sample size for main analysis....... 5

Appendix 3 Composition of the Strengths and Difficulties Questionnaire (SDQ) score in Understanding Society 6

Appendix 4 Methodology... 7

Appendix 5 Full results of parallel trends analysis 9

Appendix 6 Full results of the main analysis 10

Appendix 7 Full results of the robustness checks 11

Appendix 8 Full results of the heterogeneity effects 25

Appendix 9 Full results of the mechanism tests.. 30

References 33

Appendix 1. How does Universal Credit affect children's mental health?

Below are 7 examples of core policy changes that have been introduced in the United Kingdom that may have impacts on children mental health, as outlined in the main text (see Figure 2 in main document)

1. Two-child limit policy

Households with a third or subsequent child born after 6th April 2017, claiming Universal Credit or Child Tax Credit was no longer able to received child related amounts for these children. This came into effect for families in February 2019. As of April 2021, there were 3.69 million families with children claiming Universal Credit or Child Tax Credit(1). Among these families, 30% had three or more children. This policy change was expected to result in significant increases in the number of children living in poverty, with certain minority groups disproportionately impacted(2). The Resolution Foundation estimates that nearly half of the families with three or more children were in relative poverty in 2021/22, up from a third in 2012/13(3). The more recent research highlights the significant negative impact of two-child benefit cap policy on larger families (4) and the poorest households (5).

2. Changes in help with childcare costs for some groups

The government reduced childcare costs under UC initially, leading to a decrease in household childcare expenses from 95.5% to 70%.(6) This change resulted in parents having to pay more than six times the amount they were contributing towards their childcare costs out of their own pockets(7). However, since April 2016, UC has increased the proportion of childcare costs that can be claimed back through Universal Credit from 70% to 85%(8). Additionally, there have been several changes to accessing free childcare for 38 weeks of the year, these are detailed in the table 1 below(9).

Table 1 Description of changes to government support for childcare over time, eligibility, hours of free childcare and age of child this applies to.

Year	Eligibility	Hours*	Age of child
2010	All children	15 hours	3 and 4 year olds
2016	All children	30 hours	3 and 4 year olds
2017	Low income/in receipt of benefits	30 hours	2 year olds
April 2024	In paid work	15 hours	2 year olds
September 2024	In paid work	15 hours	9 months to 3 years
September 2025	In paid work	30 hours	9 months to 4 years

**Note: These are weekly hours of free childcare offered to parents for 38 weeks out of a year.*

3. Substantial cuts in support for disabled children

Within Child Tax Credit, families with children living with a disability were eligible for additional financial support through the disability element. This was valued at up to £53.70 per week, per child with a disability, and helped families address the supplementary needs of a child with a disability. Under Universal Credit the disability element available to parents of a child with a disability was reduced to roughly half of what was available under child tax credits. The Disability Living Allowance set at £26.9 per week in 2022/2023 for each child with a disability. The amount varies depending on the level of help the child needs.(10) The government had estimated that around 100,000 disabled children would be negatively impacted by this change(11).

4. Free School Meals and other passported benefits

Some key benefits that served as ’passporting’ criteria for entitlement to other benefits were being incorporated into Universal Credit. The government implemented of an earnings threshold to determine the eligibility criteria for Free School Meals. This threshold would have resulted in the loss of Free School Meals entitlement once earnings exceeded a certain level. This approach may have created a significant ’cliff edge’ effect, undermining the progressive work incentives inherent in the Universal Credit system. It has been estimated that a household would have needed an additional £88 per week in earnings to compensate for the loss of Free School Meals entitlement(12). In England, since 1 April 2018, a child qualified for free school meals if the parent/guardian receives Universal Credit and the household’s net income does not exceed £616.67 per month.

5. Conditionality for families with children

In the 2011 Welfare Reform Bill's “Conditionality Measures”, parents were described a work-ready once their youngest child reached 5 years. After this there was an expectation for parents to actively seek employment.

The government has increased the conditions of benefit receipt (i.e. conditionality) for families to qualify for Universal Credit. Table 2 provides an overview of the specific requirements. If the age of a children is under 1, parents are not required to seek work in order to receive Universal Credit. However, when children are 13 or above, parents are expected to spend 35 hours a week searching for employment. (13).

Table 2 The conditionality regime for the main carers of children

Under 1	Do not need to look for work in order to receive Universal Credit
Age 1	Asked to attend work-focused interviews with work coach to discuss plans for a future move into work
Age 2	Expected to take active steps to prepare for work
Age 3 or 4	Expected to work a maximum of 16 hours a week (or spend 16 hours a week looking for work)
Age 5 - 12	Expected to work a maximum of 25 hours a week (or spend 25 hours a week looking for work)
Age 13 +	Expected to work a maximum of 35 hours a week (or spend 35 hours a week looking for work)

Note: Source: Department for Work and Pensions (2017)(14)

6. The abolition of the Severe Disability Premium for adults

The Severe Disability Premium (SDP), which was worth £55.30 per week on Income Support, income-based Jobseeker’s Allowance (JSA), income-related Employment and Support Allowance (ESA) and housing benefit (i.e. legacy benefits), was paid to disabled adults who had no non-dependent adult in the household and no one else receiving Carer’s Allowance to care for them.

Under Universal Credit, SDP and Enhanced Disability Premium (EDP) has been eliminated. One particular group affected by the loss of the SDP was young carers who cared for disabled lone parents. This was because Carer’s Allowance could not be granted to children under the age of 16 who are in full-time education(15).

7. Introduction of a capital limit of £16,000

There is a capital limit of £16,000, meaning that households with savings exceeding this limit are no longer eligible for any support. These capital rules(16) have a significant impact on savers who currently receive substantial tax credit awards,

especially working parents with high childcare costs, as their tax credit award will be at risk (15).

8. Delayed benefit payments

Built into the Universal Credit claiming process, new claimants wait a minimum of five weeks to receive their first payment. This includes a minimum of 4 weeks assessment period plus 7 extra days before people receive their first payment. There is large variability in the timeframe that people wait to receive some or all of their first payment.(17) This initial wait, and associated stress has been found to heighten claimants experiences of debt, financial difficulties, increased food bank usage, and caused and exacerbated mental health difficulties. (18,19)

Appendix 2 Flowchart of the child population and sample size for main analysis

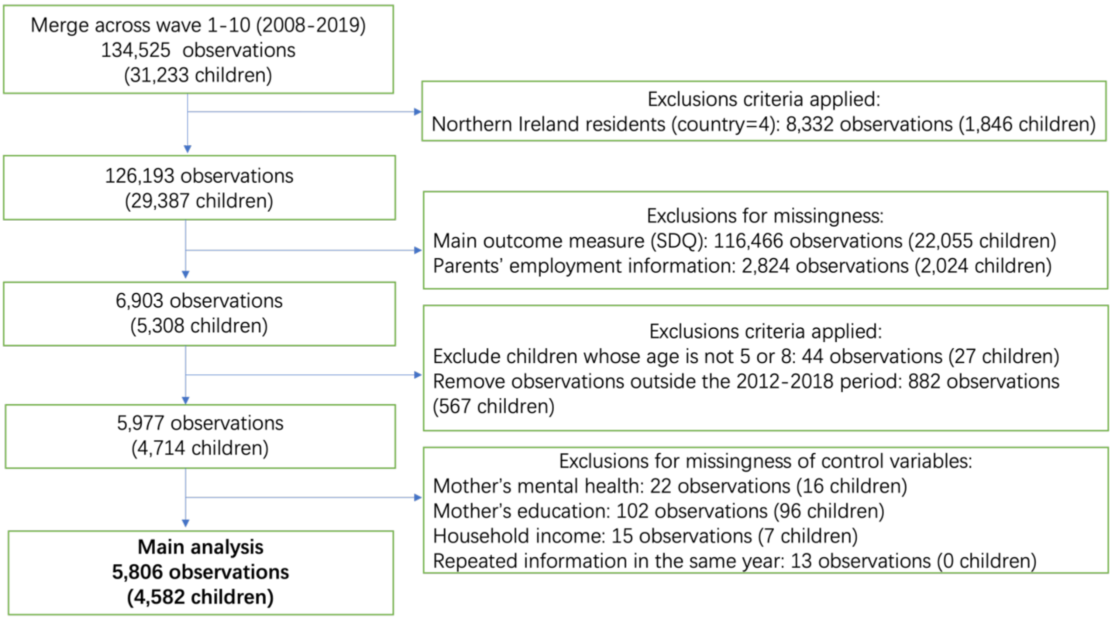

Appendix 3 Composition of the Strengths and Difficulties Questionnaire (SDQ) score in Understanding Society

Table 3 the composition of the SDQ score

Emotional problems scale (chsdqes_dv)
Often complains of headaches, stomach-aches or sickness
Many worries, often seems worried
Often unhappy, downhearted or tearful
Nervous or clingy in new situations, easily loses confidence
Many fears, easily scared
Conduct problems Scale (chsdqcp_dv)
Often has temper tantrums or hot tempers
Generally obedient, usually does what adults request
Often fights with other children or bullies them
Often lies or cheats
Steals from home, school, or elsewhere
Hyperactivity scale (chsdqha_dv)
Restless, overactive, cannot stay still for long
Constantly fidgeting or squirming
Easily distracted, concentration wanders
Thinks things out before acting
Sees tasks through to the end. good attention span
Peer problems scale (chsdqpp_dv)
Rather solitary, tends to play alone
Has at least one good friend
Generally liked by other children
Picked on or bullied by other children
Gets on better with adults than with other children
Prosocial scale (chsdqps_dv)
Considerate of other people's feelings
Shares readily with other children (treats, toys, pencils etc)
Helpful if someone is hurt, upset, or feeling ill
Kind to younger children
Often volunteers to help others (parents, teachers, other children)

Note: This table shows the composition of the SDQ score. The 25 items in the SDQ comprise 5 scales of 5 items each. Parents of 5 and 8 year olds were asked to respond in relation to their child. *The UKHLS child questionnaire provides scores for five areas, and the corresponding variable names are in parentheses. The total SDQ score(chsdqtd_dv) in the analysis is derived from the UK Household Longitudinal Study (UKHLS).*

Appendix 4 Methodology

We conducted a quantile analysis of Strengths and Difficulties Questionnaire (SDQ) scores to identify which group of children was most affected by UC. We found significant changes for children whose SDQ scores fell between the 85th and 95th percentiles, as shown in Table 4.

These results confirmed that children who were vulnerable prior to the introduction of UC experienced heightened adverse effects following its implementation. This validated the reliability of using Distress (1 if the SDQ score for children's mental health is equal to or above 17, and 0 otherwise) to categorize children into two groups in the main regression.

Table 4 Quantile Analysis of Strengths and Difficulties Questionnaire (SDQ) Scores

SDQ	Coefficient	std. err.	t	P>t	Lower 95%CI	Upper 95%CI
q20						
eligpost	-0.17	0.80	-0.22	0.83	-1.75	1.40
q40						
eligpost	-0.04	0.81	-0.05	0.96	-1.62	1.54
q60						
eligpost	2.00	1.55	1.30	0.20	-1.02	5.03
q80						
eligpost	2.23	1.69	1.32	0.19	-1.07	5.54
q85						
eligpost	3.59	1.74	2.07	0.04	0.18	6.99
q90						
eligpost	4.03	1.28	3.14	0.00	1.51	6.54
q95						
eligpost	4.35	1.79	2.43	0.02	0.84	7.87
q99						
eligpost	3.03	4.01	0.76	0.45	-4.82	10.88
Controls	YES	YES	YES	YES	YES	YES

Based on the results of the quantile analysis, we used SDQ caseness and employed the following methodology:

To estimate the treatment effect of Universal Credit on the mental health outcome $Distress_{it}$ of child i observed in year t, we employed the following regression model:

$$\log(\frac{P(distress_{it})}{1-P(distress_{it})}) = \alpha_0 + \alpha_1 Elig_{it} + \alpha_2 Elig_{it} \times Post_t + \alpha_3 X_{it} + \varepsilon_{it} \quad (1)$$

In this model, $\log(\frac{P(distress_{it})}{1-P(distress_{it})})$ represents the log odds of the probability that the outcome variable Distress_{it} equals 1. Distress_{it} is a dummy variable, taking the

value of 1 if the SDQ score for children's mental health is equal to or above 17, and 0 otherwise. The main dependent variable $Elig_{it}$ is a dummy variable that takes the value 1 if one of the children's parents was eligible for Universal Credit and 0 otherwise. The coefficient α_1 captures the differences in children's mental health between the intervention group and the comparison group in terms of their parents' Universal Credit status. The variable $Post_t$ indicates whether year t occurred after or before the intervention.

The variable of interest, $Elig_{it} \times Post_t$, represents the interaction term between the policy exposure period and the intervention group. This interaction term is set to zero in the years before Universal Credit and takes the value of one if one of the children's parents was eligible for Universal Credit after the introduction of Universal Credit for people with children in 2016.

The set of covariates X_{it} in the model includes children's age (coded as 5 or 8), children's gender (female = 0, male = 1), children's long-term health condition ("Excellent" compared with "very good", "goo", "fair" and "poor"), mothers' education ("Degree" compared with "other higher", "A levels", "GCSE", "other qualification" and "no qualification"), mothers' mental health (measured using the 12-item General Health Questionnaire (GHQ-12) score), log of household inflation-adjusted income (household income was measured as the logarithm of the contemporaneous monthly net income from the labour market and all other sources taking away any taxes, deductions, and benefits in GB 2010 prices), and whether there is just one child in the household (only one child in family = 1, additional children in family = 0). The error term ε_{it} is assumed to have a conditional mean of zero.

To analyse the causal treatment effect on mediators M_{it} (log of household income and whether the childcare service is used) for child i in period t, we estimated the benchmark equation replacing the $Distress_{it}$ with M_{it} as the dependent variable. By applying the same methodology, we can identify the causal treatment effect on each mediator.

Appendix 5 Full results of parallel trends analysis

Table 5 presented the results of the parallel trend analysis. Prior to the implementation of UC, there was no statistically significant difference between the intervention group and the comparison group. However, following the introduction of UC, the difference between the two groups became larger and statistically significant.

Table 5 Full results of parallel trends analysis

Distress	Odds ratio	Std. err.	z	P>z	Lower 95% CI	Upper 95% CI
pre_UC_4*elig	1.07	0.38	0.20	0.84	0.54	2.15
pre_UC_3*elig	0.94	0.37	-0.15	0.89	0.43	2.05
pre_UC_2*elig	1.09	0.51	0.18	0.86	0.43	2.73
pre_UC_1*elig	.	.	.	.	.	.
post_UC_0*elig	2.15	0.88	1.87	0.06	0.96	4.81
post_UC_1*elig	1.83	0.78	1.43	0.15	0.80	4.22
post_UC_2*elig	4.32	1.89	3.35	0.00	1.84	10.19
Age	1.11	0.04	2.97	0.00	1.03	1.18
Male	1.57	0.16	4.46	0.00	1.29	1.92
Mothers' mental health	1.14	0.01	10.21	0.00	1.11	1.17
Single child	0.92	0.14	-0.57	0.57	0.68	1.23
Household income	0.64	0.06	-4.50	0.00	0.52	0.78
Children's long-term health condition						
Excellent	.	.	.	.	.	.
Very good	1.81	0.21	5.02	0.00	1.44	2.28
Good	4.53	0.64	10.75	0.00	3.44	5.97
Fair	8.58	1.82	10.13	0.00	5.66	13.01
Poor	12.50	4.96	6.36	0.00	5.74	27.23
Mother's degree						
Degree	.	.	.	.	.	.
Other higher	1.21	0.22	1.08	0.28	0.85	1.73
A level etc	1.40	0.22	2.18	0.03	1.03	1.89
GCSE etc	2.14	0.30	5.43	0.00	1.63	2.82
Other qual	1.74	0.41	2.33	0.02	1.09	2.76
No qual	2.54	0.56	4.18	0.00	1.64	3.92
Constant	0.41	0.36	-1.02	0.31	0.08	2.25

Appendix 6 Full results of the main analysis

Table 6 Number of observations in the intervention and comparison groups before and after the implementation of UC

**Eligibility Approach**	**Policy Period**	**Comparison group**	**Intervention group**
Using unemployment to define eligibility	Before UC	3,396	222
	After UC	2,077	111

Table 7 Treatment effect of UC on children's mental health

Distress	Odds ratio	Std. err.	z	P>z	Lower 95%CI	Upper 95%CI
Age	1.10	0.04	2.88	0.00	1.03	1.18
Male	1.58	0.16	4.50	0.00	1.30	1.93
Mothers' mental health	1.14	0.01	10.22	0.00	1.11	1.17
Single child	0.92	0.14	-0.54	0.59	0.69	1.24
Household income	0.64	0.06	-4.45	0.00	0.53	0.78
Children's long-term health condition						
Excellent	.	.	.	.	.	.
Very good	1.79	0.21	4.93	0.00	1.42	2.25
Good	4.48	0.63	10.69	0.00	3.41	5.91
Fair	8.52	1.81	10.10	0.00	5.62	12.90
Poor	13.24	5.36	6.38	0.00	5.99	29.28
Mother's degree						
Degree	.	.	.	.	.	.
Other higher	1.23	0.22	1.17	0.24	0.87	1.75
A level etc	1.41	0.22	2.21	0.03	1.04	1.90
GCSE etc	2.17	0.30	5.50	0.00	1.64	2.85
Other qual	1.73	0.41	2.31	0.02	1.09	2.75
No qual	2.52	0.56	4.13	0.00	1.62	3.9014
elig#Post	2.18	0.72	2.34	0.02	1.14	4.18
Constant	0.39	0.34	-1.09	0.27	0.07	2.12

Table 8 Marginal test of the treatment effect

	Before and after estimator	std. err.	z	P>z	Lower 95%CI	Upper 95%CI
elig#Post (1 vs 0) (1 vs 0)	0.08	0.03	2.30	0.02	0.01	0.14

Appendix 7 Full results of the robustness checks

Table 9 shows the number of observations in the intervention and comparison groups before and after the implementation of UC in the robustness checks whenever it is different from the main sample size.

Table 9 Number of observations in the intervention and comparison groups before and after the implementation of UC in the robustness checks

**Eligibility Approach**	**Policy Period**	**Comparison group**	**Intervention group**
Recorded as in receipt of Universal Credit or one of the legacy benefits*	Before UC	3,127	491
	After UC	2,056	132
Using stable treatment status to define eligibility	Before UC	3,346	272
	After UC	2,052	136
Excluding families with more than two children	Before UC	2,255	108
	After UC	1,429	52
Dropping the highest 25% household income in the comparison group	Before UC	2,555	213
	After UC	1,549	106
Using linear probability model with individual fixed effects, including only those with more than two observations.	Before UC	1,623	84
	After UC	980	42
Using multiple imputation	Before UC	3,300	792
	After UC	1,920	213

Note: Legacy benefits include Working Tax Credit, Child Tax Credit, Housing Benefit, Income Support, income-based Jobseeker's Allowance and income-related Employment and Support Allowance (ESA).

Robustness test 1. Using receipt of UC or one of the six legacy benefits to redefine eligibility.

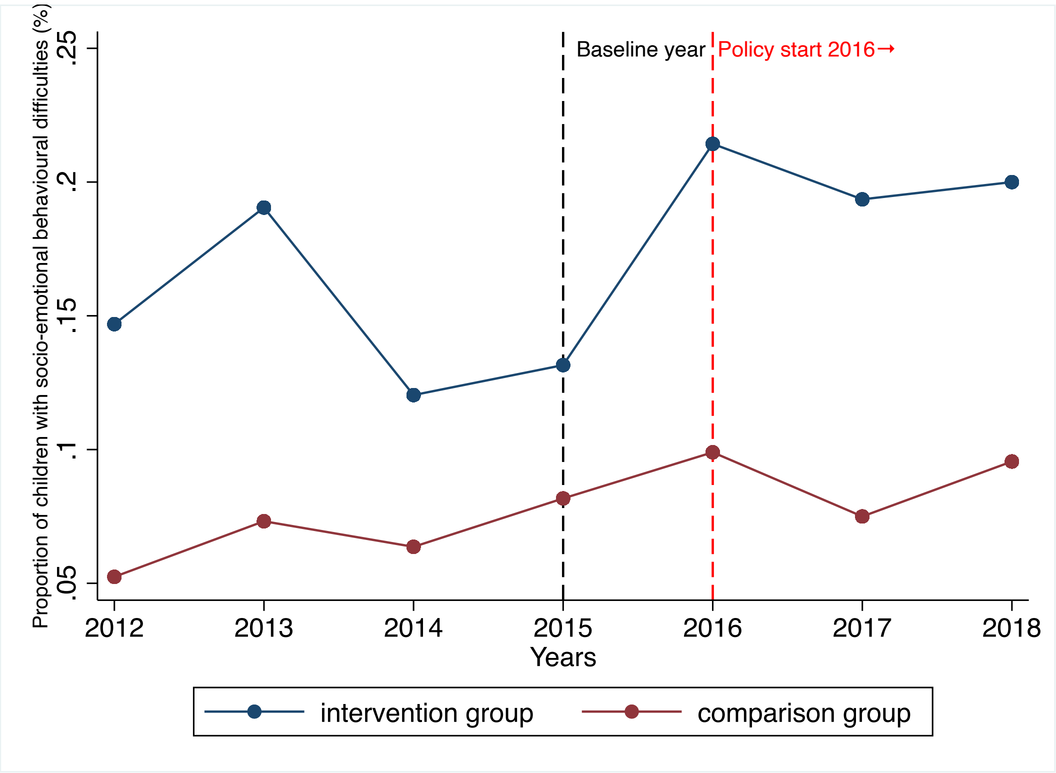

Figure 1 shows socioemotional behavioural difficulties in the intervention and comparison groups before and after Universal Credit was introduced, using receipt of UC or one of the six legacy benefits to redefine eligibility.

Table 10 Treatment effect of UC on children's mental health when using receipt of UC or one of the six legacy benefits to redefine eligibility

Distress (N= 5,806)	Odds ratio	Std. err.	z	P>z	Lower 95%CI	Upper 95%CI
Age	1.10	0.04	2.80	0.01	1.03	1.17
Male	1.59	0.16	4.53	0.00	1.30	1.94
Mothers' mental health	1.14	0.01	10.30	0.00	1.11	1.17
Single child	0.95	0.14	-0.34	0.73	0.71	1.27
Household income	0.64	0.06	-4.52	0.00	0.52	0.78
Children's long-term health condition						
Excellent	.	.	.	.	.	.
Very good	1.80	0.21	4.99	0.00	1.43	2.27
Good	4.56	0.64	10.82	0.00	3.46	6.00
Fair	8.38	1.78	10.00	0.00	5.53	12.72
Poor	12.99	5.24	6.35	0.00	5.89	28.66
Mother's degree						
Degree	.	.	.	.	.	.
Other higher	1.19	0.21	0.98	0.33	0.84	1.70
A level etc	1.38	0.21	2.09	0.04	1.02	1.87

GCSE etc	2.08	0.29	5.19	0.00	1.58	2.75
Other qual	1.77	0.42	2.44	0.02	1.12	2.80
No qual	2.37	0.53	3.87	0.00	1.53	3.68
elig#Post	1.64	0.48	1.69	0.09	0.92	2.90
Constant	0.39	0.34	-1.08	0.28	0.07	2.14

Table 11 Marginal test of the treatment effect when using receipt of UC or one of the six legacy benefits to redefine eligibility

	Before and after estimator	std. err.	z	P>z	Lower 95%CI	Upper 95%CI
elig#Post						
(1 vs 0) (1 vs 0)	0.05	0.03	1.73	0.08	-0.01	0.11

Robustness test 2. Using continuous socioemotional behavioural difficulties score as the outcome variable

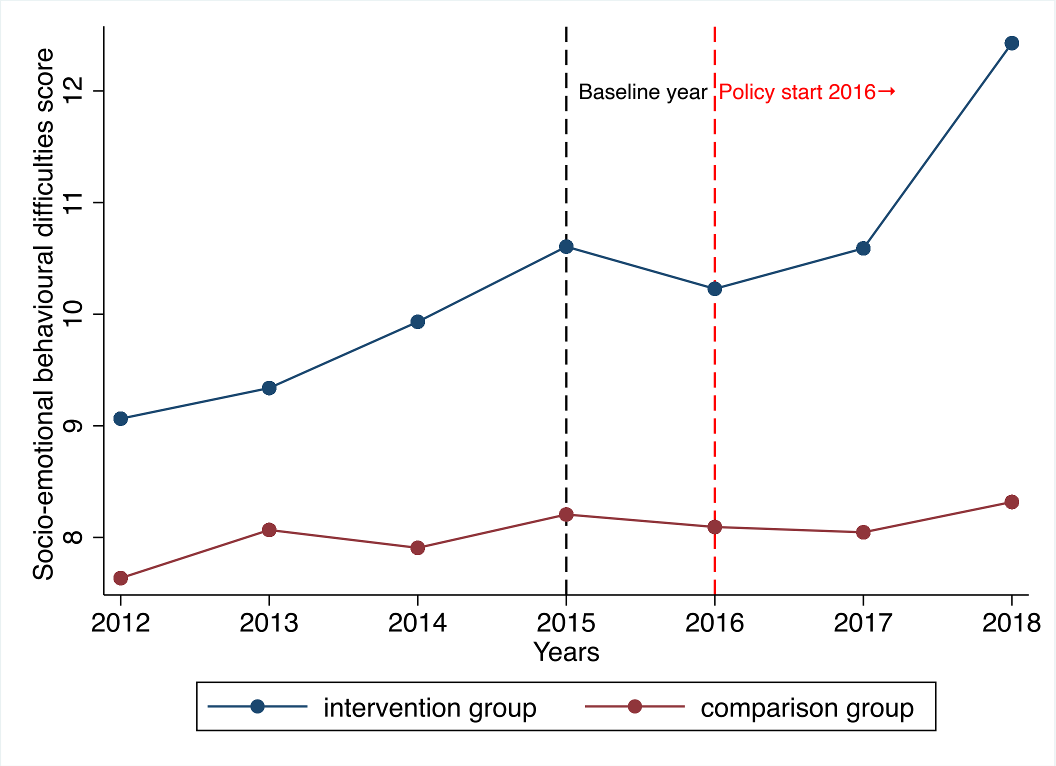

Figure 2 shows socioemotional behavioural difficulties score in the intervention and comparison groups before and after Universal Credit was introduced.

Table 12 The treatment effect when using the continuous measure of SDQ

Distress (N= 5,806)	Coefficient	Std. err.	z	P>z	Lower 95%CI	Upper 95%CI
Age	0.04	0.05	0.78	0.44	-0.05	0.12

Male	1.08	0.14	7.93	0.00	0.81	1.34
Mothers' mental health	0.35	0.02	15.72	0.00	0.31	0.39
Single child	0.02	0.20	0.08	0.94	-0.38	0.41
Household income	-0.70	0.13	-5.35	0.00	-0.95	-0.44
Children's long-term health condition						
Excellent	.	.	.	.	.	.
Very good	1.49	0.15	9.92	0.00	1.20	1.79
Good	3.63	0.25	14.69	0.00	3.15	4.12
Fair	6.13	0.46	13.26	0.00	5.22	7.03
Poor	8.00	0.97	8.28	0.00	6.11	9.90
Mother's degree						
Degree	.	.	.	.	.	.
Other higher	0.40	0.22	1.86	0.06	-0.02	0.83
A level etc	0.82	0.20	4.20	0.00	0.44	1.21
GCSE etc	1.73	0.19	8.93	0.00	1.35	2.11
Other qual	1.66	0.36	4.56	0.00	0.95	2.37
No qual	2.30	0.38	6.06	0.00	1.56	3.05
elig#Post	1.40	0.51	2.75	0.01	0.40	2.39
Constant	10.69	1.15	9.31	0.00	8.44	12.94

Robustness test 3. Using stable employment status to define eligibility

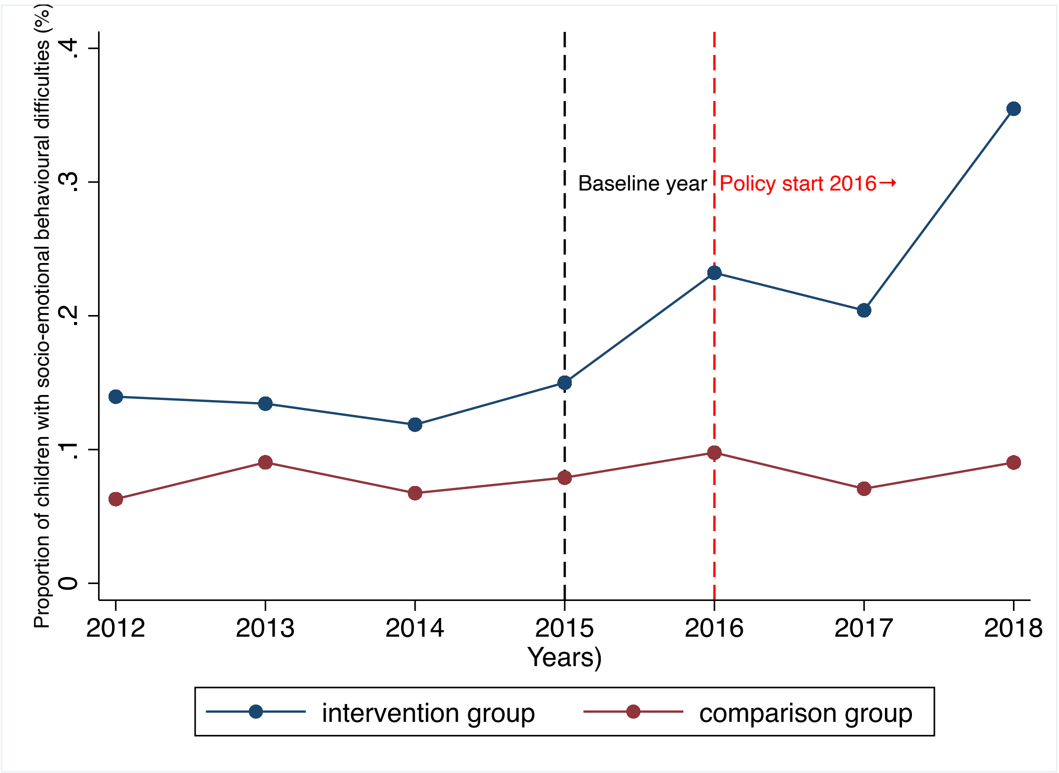

Figure 3 shows socioemotional behavioural difficulties in the intervention and comparison groups before and after Universal Credit was introduced, using stable employment status to define eligibility

Table 13 Treatment effect of UC on children's mental health when using stable employment status to define eligibility

Distress (N= 5,806)	Odds ratio	Std. err.	z	P>z	Lower 95%CI	Upper 95%CI
Age	1.10	0.04	2.78	0.01	1.03	1.17
Male	1.58	0.16	4.49	0.00	1.29	1.93
Mothers' mental health	1.14	0.01	10.21	0.00	1.11	1.17
Single child	0.92	0.14	-0.53	0.60	0.69	1.24
Household income	0.63	0.06	-4.56	0.00	0.52	0.77
Children's long-term health condition						
Excellent	.	.	.	.	.	.
Very good	1.79	0.21	4.95	0.00	1.42	2.26
Good	4.49	0.63	10.70	0.00	3.41	5.91
Fair	8.47	1.80	10.08	0.00	5.59	12.83
Poor	13.29	5.37	6.40	0.00	6.02	29.35
Mother's degree						
Degree	.	.	.	.	.	.
Other higher	1.24	0.22	1.18	0.24	0.87	1.76
A level etc	1.41	0.22	2.23	0.03	1.04	1.91
GCSE etc	2.16	0.30	5.48	0.00	1.64	2.84
Other qual	1.73	0.41	2.33	0.02	1.09	2.76

No qual	2.52	0.56	4.13	0.00	1.62	3.90
elig#Post	1.95	0.61	2.15	0.03	1.06	3.58
Constant	0.43	0.37	-0.98	0.33	0.08	2.35

Table 14 Marginal test of the treatment effect when using stable employment status to define eligibility

	Before and after estimator	std. err.	z	P>z	Lower 95%CI	Upper 95%CI
Percentage point change in prevalence of psychological distress (Distress) when using stable treatment status to define eligibility	0. 06	0.03	2.13	0.03	0.00	0.12

Robustness test 4. Excluding families with more than two children in the sample

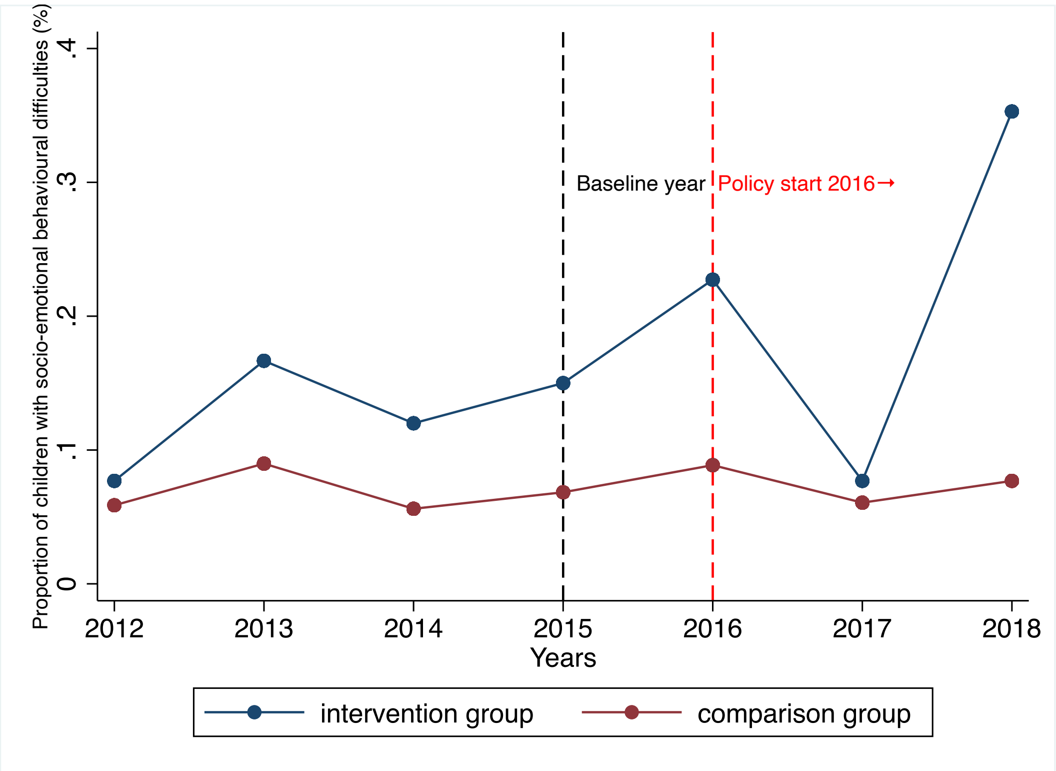

Figure 4 shows socioemotional behavioural difficulties score in the intervention and comparison groups before and after Universal Credit was introduced, when excluding families with more than two children.

Table 15 Treatment effect of UC on children's mental health when excluding households with two more children

Distress (N=3,845)	Odds ratio	Std. err.	z	P>z	Lower 95%CI	Upper 95%CI
Age	1.05	0.05	1.18	0.24	0.97	1.15
Male	1.70	0.23	4.01	0.00	1.31	2.21
Mothers' mental health	1.11	0.02	5.80	0.00	1.07	1.15

Single child	1.00	0.16	-0.01	1.00	0.73	1.36
Household income	0.61	0.08	-4.04	0.00	0.47	0.77
Children's long-term health condition						
Excellent	.	.	.	.	.	.
Very good	1.78	0.27	3.85	0.00	1.33	2.39
Good	4.85	0.89	8.62	0.00	3.39	6.94
Fair	7.61	2.22	6.95	0.00	4.30	13.50
Poor	7.64	4.13	3.76	0.00	2.65	22.04
Mother's degree						
Degree	.	.	.	.	.	.
Other higher	1.05	0.24	0.24	0.81	0.68	1.64
A level etc	1.53	0.28	2.30	0.02	1.06	2.19
GCSE etc	1.90	0.34	3.55	0.00	1.33	2.71
Other qual	1.49	0.48	1.23	0.22	0.79	2.79
No qual	1.99	0.65	2.08	0.04	1.04	3.79
elig#Post	1.68	0.85	1.02	0.31	0.62	4.50
Constant	0.87	0.94	-0.13	0.90	0.11	7.15

Table 16 Marginal test of the treatment effect when using excluding households with two more children

	Before and after estimator	std. err.	z	P>z	Lower 95%CI	Upper 95%CI
Percentage point change in prevalence of psychological distress (Distress) when excluding households with two more children	0.04	0.04	1.00	0.32	-0.04	0.12

Robustness test 5. Dropping the highest 25% household income in the comparison group

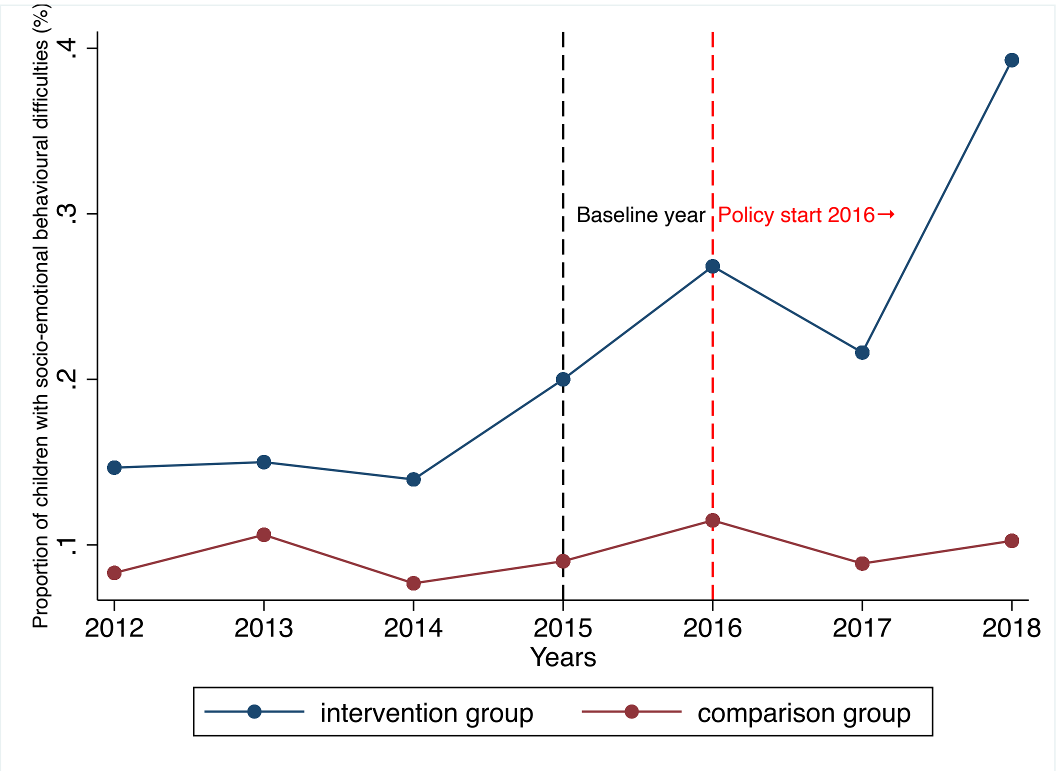

Figure 5 shows socioemotional behavioural difficulties score in the intervention and comparison groups before and after Universal Credit was introduced, when dropping the highest 25% household income in the comparison group.

Table 17 Treatment effect of UC on children's mental health when dropping the highest 25% household income in the comparison group.

Distress (N=4,425)	Odds ratio	Std. err.	z	P>z	Lower 95%CI	Upper 95%CI
Age	1.11	0.04	2.86	0.00	1.03	1.19
Male	1.65	0.18	4.59	0.00	1.33	2.04
Mothers' mental health	1.15	0.02	9.93	0.00	1.12	1.18
Single child	1.05	0.17	0.31	0.75	0.77	1.43
Household income	0.76	0.10	-2.08	0.04	0.59	0.98
Children's long-term health condition						
Excellent	.	.	.	.	.	.
Very good	1.86	0.24	4.88	0.00	1.45	2.39
Good	4.88	0.73	10.68	0.00	3.65	6.53
Fair	9.57	2.16	10.02	0.00	6.15	14.89
Poor	13.97	6.17	5.97	0.00	5.88	33.18
Mother's degree						
Degree	.	.	.	.	.	.
Other higher	1.33	0.26	1.43	0.15	0.90	1.96
A level etc	1.50	0.26	2.36	0.02	1.07	2.09
GCSE etc	2.36	0.36	5.55	0.00	1.74	3.19
Other qual	1.88	0.46	2.56	0.01	1.16	3.04

No qual	2.76	0.64	4.36	0.00	1.75	4.36
elig#Post	2.18	0.74	2.29	0.02	1.12	4.24
Constant	0.08	0.09	-2.24	0.03	0.01	0.74

Table 18 Marginal test of the treatment effect when dropping the highest 25% household income in the comparison group.

	Before and after estimator	std. err.	z	P>z	Lower 95%CI	Upper 95%CI
Percentage point change in prevalence of psychological distress (Distress) when excluding 25% of households with the highest income	0.09	0.04	2.29	0.02	0.01	0.16

Robustness test 6. Using linear probability model with individual fixed effects, including only children with more than two observations

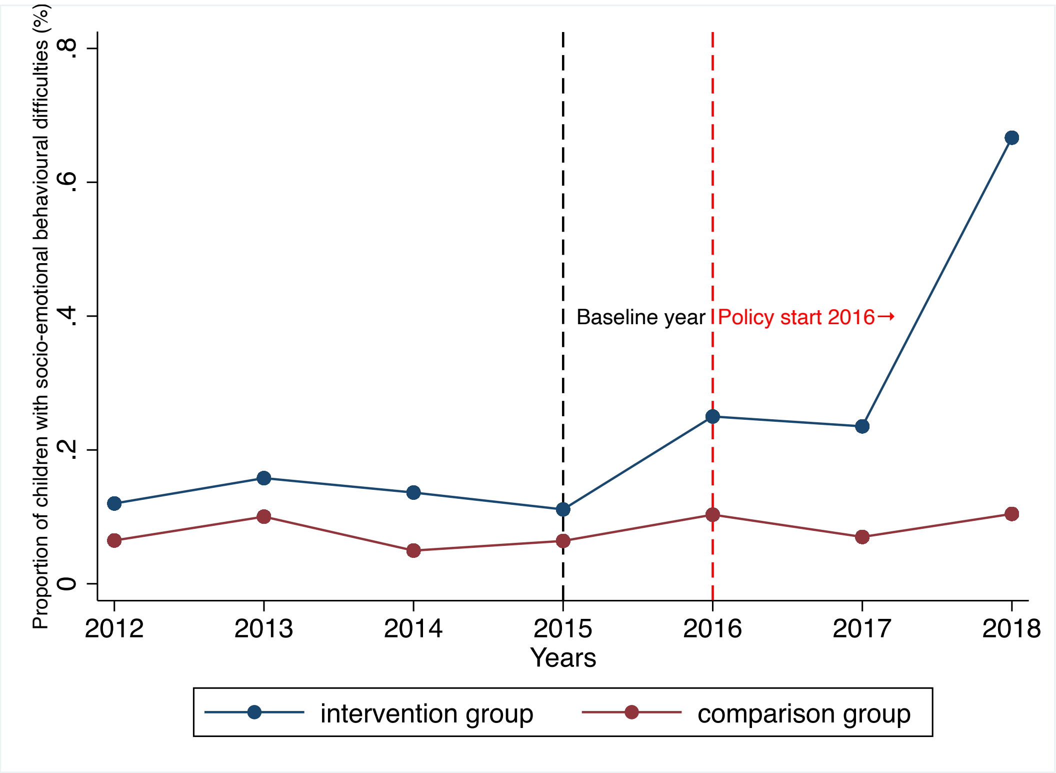

Figure 6 shows socioemotional behavioural difficulties score in the intervention and comparison groups before and after Universal Credit was introduced, using linear probability model with individual fixed effects and including only those with more than two observations.

Table 19 Treatment effect of UC on children's mental health when using linear probability model with individual fixed effects, including only those with more than two observations

Distress (N=2,729)	Coefficient	Std. err.	z	P>z	Lower 95%CI	Upper 95%CI

Age	0.00	0.01	-0.62	0.54	-0.01	0.01
Male	(omitted)					
Mothers' mental health	0.00	0.00	0.80	0.42	0.00	0.01
Single child	0.04	0.04	1.24	0.22	-0.03	0.11
Household income	-0.02	0.02	-1.15	0.25	-0.06	0.01
Children's long-term health condition						
Excellent	.	.	.	.	.	.
Very good	0.03	0.02	1.66	0.10	-0.01	0.06
Good	0.13	0.03	4.49	0.00	0.07	0.18
Fair	0.20	0.05	3.61	0.00	0.09	0.30
Poor	0.42	0.10	4.38	0.00	0.23	0.61
Mother's degree						
Degree	.	.	.	.	.	.
Other higher	-0.11	0.09	-1.15	0.25	-0.29	0.08
A level etc	-0.08	0.14	-0.59	0.55	-0.36	0.19
GCSE etc	-0.07	0.14	-0.50	0.62	-0.34	0.20
Other qual	-0.04	0.14	-0.28	0.78	-0.31	0.23
No qual	-0.39	0.21	-1.86	0.06	-0.80	0.02
elig#Post	0.14	0.06	2.47	0.01	0.03	0.26
Constant	0.28	0.16	1.74	0.08	-0.04	0.60

Robustness test 7. PSM score matching

Table 20 and Table 21 showed the matching quality. After propensity score matching (PSM) matching, the intervention and control groups were statistically similar, reducing the differences between them. Table 23 estimated the marginal impact of Universal Credit using two different matching methods: Radius and Kernel matching. The results indicated that the prevalence of psychological distress in the intervention group increased by approximately 11 percentage points following the introduction of UC, compared to the comparison group.

Table 20 Balancing Test After PSM Matching

	Unmatched	Mean			%reduct	t-test		V(T)/
Variable	Matched	Treated	Control	%bias	bias	t	p>t	V(C)
Age	U	6.54	6.48	3.50		0.62	0.53	1.00
	M	6.54	6.46	4.80	-36.70	0.62	0.54	1.00
Male	U	0.50	0.51	-2.00		-0.35	0.73	.
	M	0.50	0.48	3.60	-84.90	0.47	0.64	.
Mothers' mental health	U	2.71	1.79	27.40		5.28	0.00	1.44*

	M	2.71	2.37	10.00	63.50	1.18	0.24	0.99
Single child	U	0.10	0.13	-10.80		-1.80	0.07	.
	M	0.10	0.08	3.80	64.60	0.54	0.59	.
Household income	U	7.66	8.23	-111.80		-18.29	0.00	0.68*
	M	7.66	7.67	-2.90	97.40	-0.40	0.69	0.82
Children's long-term health condition								
Very good	U	0.32	0.32	-0.10		-0.01	0.99	.
	M	0.32	0.32	0.60	-932.60	0.08	0.93	.
Good	U	0.14	0.09	16.00		3.12	0.00	.
	M	0.14	0.14	0.00	100.00	0.00	1.00	.
Fair	U	0.04	0.02	10.30		2.10	0.04	.
	M	0.04	0.05	-7.00	31.50	-0.75	0.46	.
Poor	U	0.01	0.00	5.20		1.08	0.28	.
	M	0.01	0.02	-10.90	-110.90	-1.01	0.32	.
Mother's degree								
Other higher	U	0.07	0.14	-21.50		-3.41	0.00	.
	M	0.07	0.06	3.00	86.20	0.46	0.64	.
A level etc	U	0.14	0.19	-14.20		-2.38	0.02	.
	M	0.14	0.13	3.20	77.30	0.45	0.65	.
GCSE etc	U	0.35	0.20	32.70		6.28	0.00	.
	M	0.35	0.36	-1.40	95.80	-0.16	0.87	.
Other qual	U	0.10	0.04	25.20		5.71	0.00	.
	M	0.10	0.11	-4.80	80.90	-0.50	0.61	.
No qual	U	0.14	0.03	39.30		10.13	0.00	.
	M	0.14	0.14	0.00	100.00	0.00	1.00	.

Table 21 Differences between intervention and comparison groups before and after PSM matching

Sample	Ps R2	LR chi2	p>chi2	Mean Bias	Med Bias	B	R	%Var
Unmatched	0.167	423.8	0	22.8	15.1	125.2*	0.84	67
Matched	0.005	4.71	0.989	4	3.4	16.8	0.95	0

Table 22 Treatment effect of UC on children's mental health when using different matching methods with bootstrapping

Distress	Odds ratio	Std. err.	z	P>z	Lower 95%CI	Upper 95%CI
Kernel matching	1.82	0.59	1.84	0.07	0.96	3.42
Radius matching	1.81	0.55	1.96	0.05	1.00	3.28

Table 23 Marginal test of the treatment effect after different matching methods with bootstrapping

	Before and after estimator	std. err.	z	P>z	Lower 95%CI	Upper 95%CI
Percentage point change in prevalence of difficulties for kernel matching	0.11	0.05	2.05	0.04	0.00	0.21
Percentage point change in prevalence of difficulties for for radius matching	0.11	0.05	2.26	0.02	0.01	0.21

Robustness test 8. Using linear ramp function to re-estimate the result

Table 24 Treatment effect of UC on children's mental health when using linear ramp function

Distress (N=5,806)	Odds ratio	Std. err.	z	P>z	Lower 95%CI	Upper 95%CI
Age	1.10	0.04	2.92	0.00	1.03	1.18
Male	1.57	0.16	4.44	0.00	1.29	1.92
Mothers' mental health	1.14	0.01	10.24	0.00	1.11	1.17
Single child	0.92	0.14	-0.56	0.58	0.69	1.23
Household income	0.63	0.06	-4.62	0.00	0.52	0.77
Children's long-term health condition						
Excellent	.	.	.	.	.	.
Very good	1.81	0.21	5.04	0.00	1.44	2.28
Good	4.52	0.63	10.76	0.00	3.44	5.95
Fair	8.55	1.81	10.13	0.00	5.65	12.95
Poor	12.56	4.98	6.39	0.00	5.78	27.32
Mother's degree						
Degree	.	.	.	.	.	.
Other higher	1.21	0.22	1.07	0.28	0.85	1.72
A level etc	1.40	0.22	2.17	0.03	1.03	1.89
GCSE etc	2.15	0.30	5.45	0.00	1.63	2.82
Other qual	1.72	0.41	2.28	0.02	1.08	2.73
No qual	2.56	0.57	4.26	0.00	1.66	3.95
elig_post_t	1.57	0.18	3.88	0.00	1.25	1.97
Constant	0.44	0.37	-0.97	0.33	0.08	2.33

Table 25 Marginal test of the treatment effect when using linear ramp function

	Before and after estimator	std. err.	z	P>z	Lower 95%CI	Upper 95%CI
Percentage point change in prevalence of psychological distress (Distress) when using linear ramp function	0.03	0.01	3.87	0.00	0.02	0.05

Robustness test 9. Analysis of the missing values

Firstly, we used multiple imputation to fill in observations with missing SDQ scores while retaining other information. presented the marginal results using multiple imputation. The difference in terms of the effect of treatment was not substantial after multiple imputation, which demonstrated the robustness of the results.

Table 26 Treatment effect of UC on children's mental health when using multiple imputation

Distress (N=6,225)	Odds ratio	Std. err.	z	P>z	Lower 95%CI	Upper 95%CI
Age	0.10	0.03	2.93	0.00	0.03	0.16
Male	0.45	0.10	4.39	0.00	0.25	0.65
Mothers' mental health	0.13	0.01	10.09	0.00	0.11	0.16
Single child	-0.08	0.15	-0.55	0.58	-0.38	0.21
Household income	-0.44	0.10	-4.41	0.00	-0.64	-0.25
Children's long-term health condition						
Excellent	.	.	.	.	.	.
Very good	0.57	0.12	4.81	0.00	0.34	0.80
Good	1.47	0.14	10.49	0.00	1.20	1.75
Fair	2.11	0.21	9.98	0.00	1.70	2.53
Poor	2.56	0.40	6.32	0.00	1.76	3.35
Mother's degree						
Degree	.	.	.	.	.	.
Other higher	0.21	0.18	1.19	0.23	-0.14	0.57
A level etc	0.33	0.15	2.13	0.03	0.03	0.63
GCSE etc	0.75	0.14	5.36	0.00	0.48	1.03
Other qual	0.53	0.24	2.23	0.03	0.06	0.99
No qual	0.91	0.22	4.06	0.00	0.47	1.35
elig*post	0.79	0.33	2.37	0.02	0.14	1.44
Constant	-0.94	0.87	-1.08	0.28	-2.65	0.77

Table 27 Marginal test of the treatment effect when using multiple imputation

	Before and after estimator	std. err.	Lower 95%CI	Upper 95%CI
elig#Post				
(1 vs 0) (1 vs 0)	0.08	0.04	0.01	0.15

Secondly, we used inverse probability weighting to address the missing values, providing more accurate and unbiased estimates by assigning weights to each

observation based on the probability of its inclusion. After applying the weights, the effect size increased, highlighting stronger effects that were previously attenuated by missing data. This analysis accounted for potential selection bias and can be seen in Table 29.

Table 28 Treatment effect of UC on children’s mental health when using inverse probability weighting

Distress (N=5,806)	Odds ratio	Std. err.	z	P>z	Lower 95%CI	Upper 95%CI
Age	0.99	0.04	-0.20	0.84	0.91	1.08
Male	1.09	0.14	0.70	0.48	0.85	1.41
Mothers’ mental health	1.01	0.02	0.89	0.38	0.98	1.04
Single child	0.98	0.19	-0.11	0.92	0.67	1.43
Household income	1.05	0.14	0.41	0.68	0.82	1.36
Children's long-term health condition						
Excellent	.	.	.	.	.	.
Very good	1.04	0.14	0.32	0.75	0.80	1.37
Good	0.94	0.14	-0.43	0.67	0.69	1.27
Fair	1.08	0.25	0.32	0.75	0.68	1.69
Poor	0.93	0.37	-0.18	0.85	0.43	2.02
Mother's degree						
Degree	.	.	.	.	.	.
Other higher	0.81	0.17	-0.97	0.33	0.54	1.23
A level etc	1.02	0.19	0.12	0.90	0.71	1.47
GCSE etc	0.80	0.13	-1.35	0.18	0.59	1.10
Other qual	0.82	0.22	-0.75	0.45	0.48	1.38
No qual	0.81	0.20	-0.87	0.38	0.50	1.30
elig*Post	2.98	1.18	2.76	0.01	1.37	6.46
Constant	0.69	0.76	-0.34	0.73	0.08	6.01

Table 29 Marginal test of the treatment effect using inverse probability weighting

	Before and after estimator	std. err.	z	P>z	Lower 95%CI	Upper 95%CI
Percentage point change in prevalence of psychological distress (Distress) when using inverse probability weighting	0.25	0.09	2.82	0.01	0.08	0.42

Appendix 8 Full results of the heterogeneity effects

1. The impact of UC on age specific effects

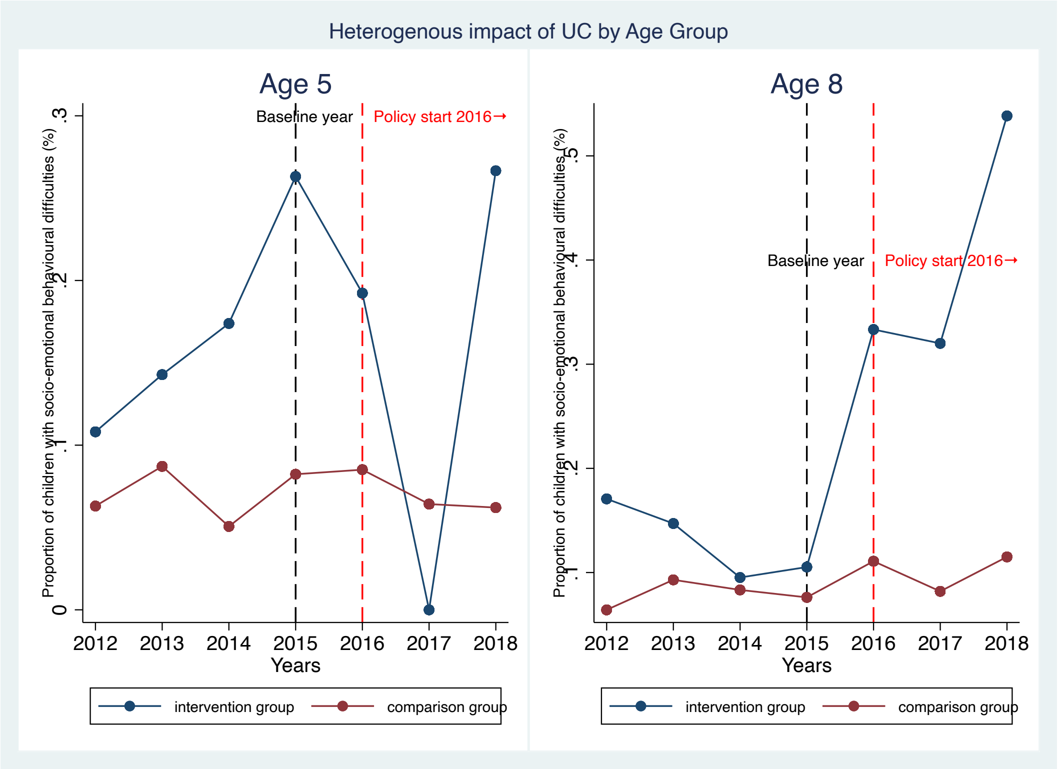

Figure 7 shows socio-emotional behavioral difficulties scores in the intervention and comparison groups before and after Universal Credit was introduced for two groups: children aged 5 and children aged 8. Note: It indicates that for children aged 8, there is a significant increase in the proportion of children with socio-emotional behavioral difficulties.

Table 30 Treatment effect of UC on children's mental health for children's aged 8

Distress	Odds ratio	Std. err.	z	P>z	Lower 95%CI	Upper 95%CI
Male	1.60	0.22	3.36	0.00	1.22	2.10
Mothers' mental health	1.16	0.02	8.76	0.00	1.13	1.20
Single child	0.77	0.17	-1.18	0.24	0.50	1.18
Household income	0.63	0.09	-3.44	0.00	0.48	0.82
Children's long-term health condition						
Excellent	.	.	.	.	.	.
Very good	1.64	0.26	3.07	0.00	1.20	2.24
Good	4.28	0.82	7.62	0.00	2.94	6.21
Fair	7.88	2.55	6.39	0.00	4.18	14.84
Poor	19.66	11.83	4.95	0.00	6.04	63.97
Mother's degree						
Degree	.	.	.	.	.	.

Other higher	1.29	0.31	1.09	0.28	0.81	2.06
A level etc	1.47	0.31	1.81	0.07	0.97	2.22
GCSE etc	1.95	0.38	3.39	0.00	1.33	2.87
Other qual	1.50	0.49	1.23	0.22	0.79	2.85
No qual	2.52	0.76	3.06	0.00	1.39	4.54
elig*Post	4.15	1.90	3.11	0.00	1.69	10.17
Constant	0.96	1.11	-0.03	0.97	0.10	9.27

Table 31 Marginal test of the treatment effect for children aged 8

	Before and after estimator	std. err.	z	P>z	Lower 95%CI	Upper 95%CI
Percentage point change in prevalence of psychological distress (Distress) for children aged 8	0.16	0.05	2.92	0.00	0.05	0.26

Table 32 Treatment effect of UC on children's mental health for children's aged 5

Distress	Odds ratio	Std. err.	z	P>z	Lower 95%CI	Upper 95%CI
Male	1.57	0.24	2.99	0.00	1.17	2.11
Mothers' mental health	1.12	0.02	5.54	0.00	1.07	1.16
Single child	1.10	0.23	0.47	0.64	0.73	1.66
Household income	0.65	0.10	-2.90	0.00	0.48	0.87
Children's long-term health condition						
Excellent	.	.	.	.	.	.
Very good	1.97	0.35	3.86	0.00	1.40	2.79
Good	4.55	0.96	7.20	0.00	3.01	6.87
Fair	9.26	2.63	7.85	0.00	5.31	16.15
Poor	8.05	4.75	3.53	0.00	2.53	25.60
Mother's degree						
Degree	.	.	.	.	.	.
Other higher	1.11	0.31	0.36	0.72	0.64	1.92
A level etc	1.31	0.30	1.17	0.24	0.83	2.04
GCSE etc	2.44	0.49	4.41	0.00	1.64	3.62
Other qual	2.08	0.72	2.13	0.03	1.06	4.08
No qual	2.70	0.91	2.95	0.00	1.40	5.22
elig*Post	0.99	0.51	-0.01	0.99	0.36	2.72
Constant	0.59	0.74	-0.42	0.67	0.05	6.93

Table 33 Marginal test of the treatment effect for children aged 5

	Before and after estimator	std. err.	z	P>z	Lower 95%CI	Upper 95%CI
Percentage point change in prevalence of psychological distress (Distress) for children aged 5	-0.00	0.04	-0.03	0.98	-0.08	0.08

2. The impact of UC on household size specific effects

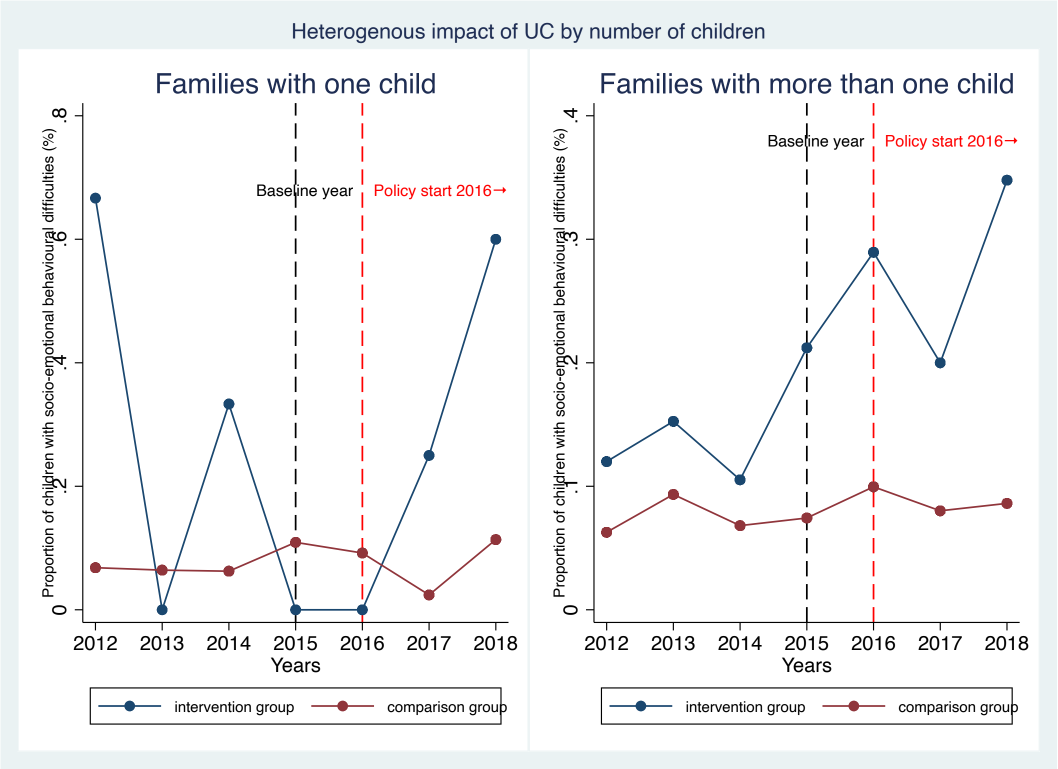

Figure 8 shows socio-emotional behavioral difficulties scores in the intervention and comparison groups before and after Universal Credit was introduced for two groups: families with one child and families with more than one child.

Note: It indicates that for larger families, there is a significant increase in the proportion of children with socio-emotional behavioral difficulties.

Table 34 Treatment effect of UC on children's mental health for one-child family

Distress	Odds ratio	Std. err.	z	P>z	Lower 95%CI	Upper 95%CI
Age	1.03	0.10	0.33	0.74	0.85	1.25
Male	2.29	0.72	2.63	0.01	1.24	4.24
Mothers' mental health	1.09	0.04	2.17	0.03	1.01	1.18
Household income	0.32	0.09	-3.95	0.00	0.18	0.56

Children's long-term health condition						
Excellent	.	.	.	.	.	.
Very good	1.98	0.68	1.99	0.05	1.01	3.89
Good	5.18	2.15	3.97	0.00	2.30	11.66
Fair	15.75	9.91	4.38	0.00	4.59	54.05
Poor	7.87	10.84	1.50	0.13	0.53	117.22
Mother's degree						
Degree	.	.	.	.	.	.
Other higher	0.98	0.49	-0.05	0.96	0.36	2.62
A level etc	0.84	0.38	-0.38	0.71	0.34	2.061817
GCSE etc	1.44	0.58	0.91	0.36	0.66	3.17
Other qual	2.41	1.37	1.55	0.12	0.79	7.37
No qual	0.79	0.69	-0.27	0.79	0.14	4.37
Elig*post	1.12	1.12	0.11	0.91	0.16	7.90
Constant	174.92	426.10	2.12	0.03	1.48	20714.72

Table 35 Marginal test of the treatment effect for one-child family

	Before and after estimator	std. err.	z	P>z	Lower 95%CI	Upper 95%CI
Percentage point change in prevalence of psychological distress (Distress) for one-child family	0.01	0.08	0.09	0.93	-0.15	0.16

Table 36 Treatment effect of UC on children's mental health for family more than one child

Distress	Odds ratio	Std. err.	z	P>z	Lower 95%CI	Upper 95%CI
Age	1.12	0.04	3.03	0.00	1.04	1.20
Male	1.50	0.16	3.75	0.00	1.21	1.86
Mothers' mental health	1.15	0.02	10.06	0.00	1.12	1.18
Household income	0.72	0.08	-3.09	0.00	0.58	0.89
Children's long-term health condition						
Excellent	.	.	.	.	.	.
Very good	1.75	0.22	4.46	0.00	1.37	2.25
Good	4.48	0.67	9.97	0.00	3.34	6.02
Fair	8.09	1.84	9.20	0.00	5.18	12.63
Poor	12.01	5.03	5.93	0.00	5.28	27.30
Mother's degree						
Degree	.	.	.	.	.	.

Other higher	1.28	0.25	1.29	0.20	0.88	1.87
A level etc	1.55	0.26	2.68	0.01	1.13	2.15
GCSE etc	2.33	0.35	5.60	0.00	1.73	3.12
Other qual	1.53	0.41	1.58	0.11	0.90	2.59
No qual	2.91	0.68	4.57	0.00	1.84	4.61
elig*post	2.40	0.86	2.46	0.01	1.20	4.83
Constant	0.14	0.13	-2.11	0.04	0.02	0.87

Table 37 Marginal test of the treatment effect for family with more than one child

	Before and after estimator	std. err.	z	P>z	Lower 95%CI	Upper 95%CI
Percentage point change in prevalence of psychological distress (Distress) for families more than one child	0.09	0.04	2.40	0.02	0.02	0.16

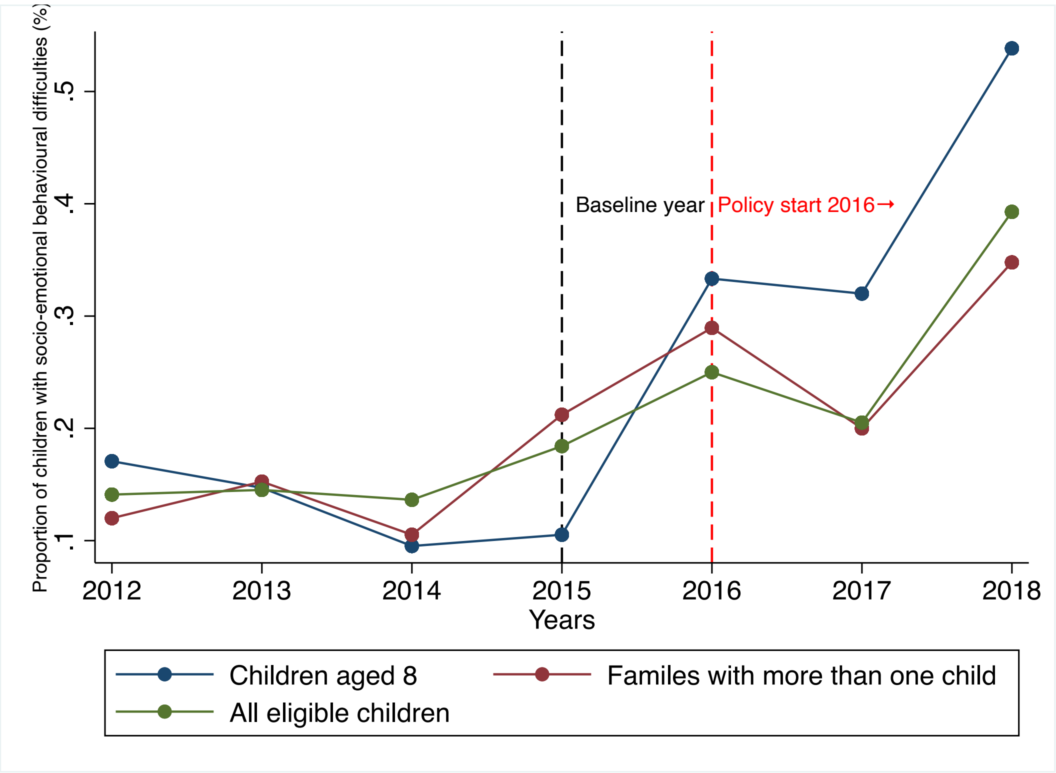

Figure 9 shows socioemotional behavioural difficulties in the intervention group before and after Universal Credit was introduced.

Note: The graph indicates a significant change for all eligible children, children aged 8, and children living in households with more than one child.

Appendix 9 Full results of the mechanism tests

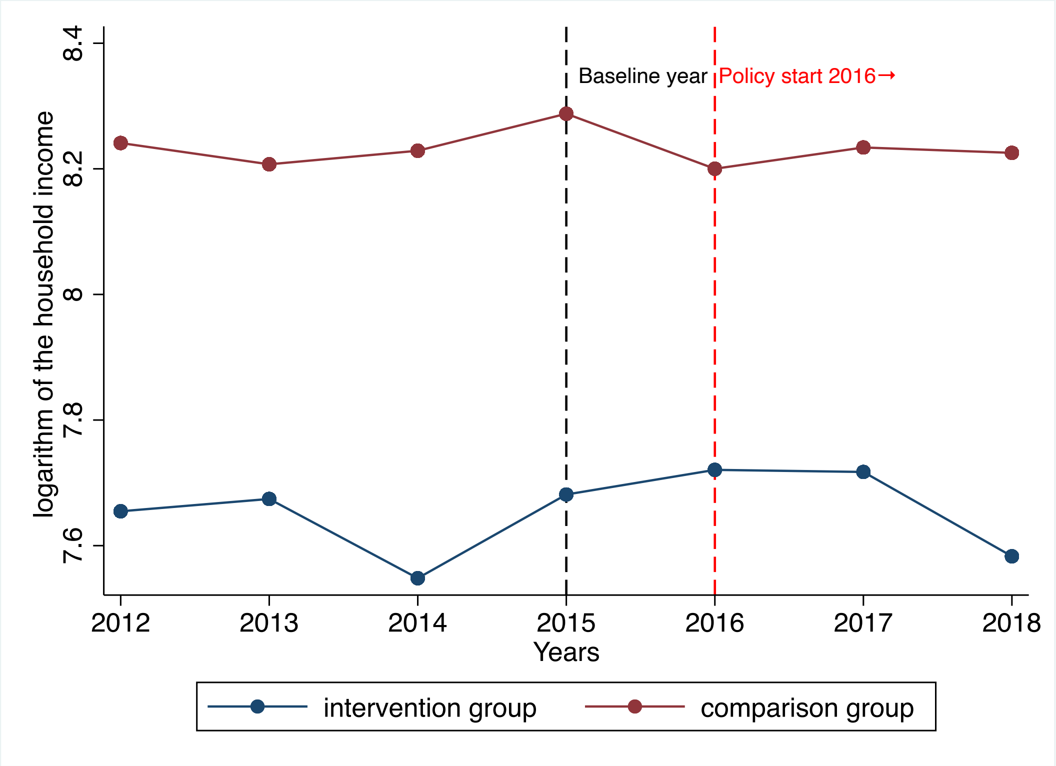

Figure 10 shows the logarithm of the household income in the intervention and comparison groups before and after Universal Credit was introduced.

Table 38 The treatment effect of UC on children's mental health using the logarithm of household income as a mediator

Household income	Coefficient	Std. err.	z	P>z	Lower 95%CI	Upper 95%CI
Age	0.01	0.00	2.52	0.01	0.00	0.02
Male	0.00	0.01	0.04	0.96	-0.03	0.03
Mothers' mental health	-0.01	0.00	-3.43	0.00	-0.01	0.00
Single child	0.01	0.02	0.59	0.56	-0.03	0.05
Children's long-term health condition						
Excellent	.	.	.	.	.	.
Very good	-0.03	0.02	-2.21	0.03	-0.06	0.00
Good	-0.11	0.02	-4.28	0.00	-0.16	-0.06
Fair	-0.16	0.05	-3.53	0.00	-0.26	-0.07
Poor	-0.28	0.10	-2.89	0.00	-0.47	-0.09
Mother's degree						
Degree	.	.	.	.	.	.
Other higher	-0.22	0.02	-10.27	0.00	-0.27	-0.18
A level etc	-0.35	0.02	-18.53	0.00	-0.39	-0.32
GCSE etc	-0.44	0.02	-23.39	0.00	-0.47	-0.40

Other qual	-0.47	0.04	-12.94	0.00	-0.54	-0.40
Other higher	-0.55	0.04	-14.72	0.00	-0.63	-0.48
No qual	-0.55	0.04	-14.71	0.00	-0.63	-0.48
elig#Post	0.06	0.06	0.91	0.36	-0.07	0.18
Constant	8.43	0.03	253.77	0.00	8.37	8.50

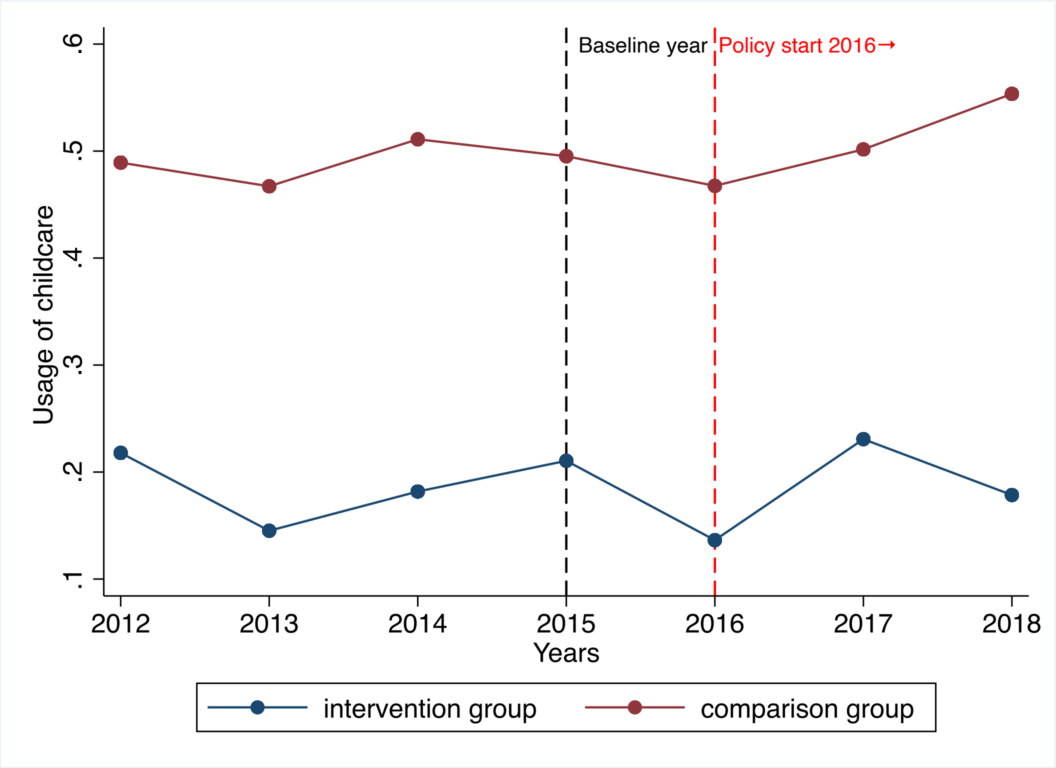

Figure 11 shows the usage of childcare service in the intervention and comparison groups before and after Universal Credit was introduced.

Table 39 The treatment effect of UC on children's mental health using the utilization of childcare services as a mediator

Childcare	Odds ratio	Std. err.	z	P>z	Lower 95%CI	Upper 95%CI
Age	0.94	0.02	-3.30	0.00	0.90	0.97
Male	0.99	0.06	-0.11	0.91	0.89	1.11
Mothers' mental health	1.02	0.01	1.95	0.05	1.00	1.04
Single child	1.28	0.11	2.87	0.00	1.08	1.51
Household income	2.79	0.17	16.51	0.00	2.47	3.15
Children's long-term health condition						
Excellent	.	.	.	.	.	.
Very good	0.87	0.06	-2.17	0.03	0.77	0.99
Good	0.80	0.08	-2.11	0.04	0.65	0.98

Fair	0.60	0.12	-2.49	0.01	0.40	0.90
Poor	0.34	0.17	-2.19	0.03	0.13	0.89
Mother's degree						
Degree	.	.	.	.	.	.
Other higher	0.77	0.07	-2.89	0.00	0.65	0.92
A level etc	0.59	0.05	-6.67	0.00	0.50	0.69
GCSE etc	0.41	0.03	-11.06	0.00	0.35	0.48
Other qual	0.27	0.05	-7.56	0.00	0.20	0.38
Other higher	0.13	0.03	-8.94	0.00	0.08	0.20
No qual	0.13	0.03	-8.94	0.00	0.08	0.20
elig#Post	0.74	0.24	-0.91	0.36	0.39	1.41
Constant	0.00	0.00	-14.26	0.00	0.00	0.00

Table 40 Marginal test of the treatment effect when using the utilization of childcare services as a mediator

	Before and after estimator	std. err.	z	P>z	Lower 95%CI	Upper 95%CI
elig#Post						
(1 vs 0) (1 vs 0)	-0.06	0.03	-0.93	0.35	-0.18	0.06

References

1. Hobson BF. The impact of the two-child limit in Universal Credit. 2022;(April):1–24.

2. Machin R. The Professional and Ethical Dilemmas of the Two-child Limit for Child Tax Credit and Universal Credit. Ethics Soc Welf [Internet]. 2017;11(4):404–11. Available from: https://doi.org/10.1080/17496535.2017.1386227

3. Browne J, Hood A. Living standards, poverty and inequality in the UK: 2015-2016 to 2020-2021. 2016.

4. Stewart K. The two-child limit: a growing hole in the UK's safety net. The London School of Economics and Politcal Science [Internet]. 2023; Available from: https://www.lse.ac.uk/research/research-for-the-world/politics/two-child-benefit-cap-poverty

5. Latimer E, Waters T. The two-child limit: poverty, incentives and cost. 2024.

6. Citzens Advice Bureau. Childcare costs in Universal Credit. 2013.

7. Royston S. Understanding universal credit. J Poverty Soc Justice. 2012;20(1):69–86.

8. Child Poverty Action Group. Broken Promises: What has Happened to Support for Low-income Working Damilies Under Universal Credit? 2017;(March):1–28. Available from: http://www.cpag.org.uk/sites/default/files/Broken promises FINAL for website.pdf

9. The Guardian. Budget 2023: Hunt overhauls pensions, benefits and childcare in push for growth [Internet]. 2023. Available from: https://www.theguardian.com/uk-news/2023/mar/15/budget-2023-jeremy-hunt-says-uk-will-avoid-recession-this-year

10. GOV.UK. Disability Living Allowance (DLA) for children [Internet]. Available from: https://www.gov.uk/disability-living-allowance-children

11. Graylin C. In response to a parliamentary question [Internet]. 2011. Available from: https://www.theyworkforyou.com/wrans/?id=2011-06-08c.57941.h&s=curran+section%3Awrans+section%3Awms#g57941.q0

12. Great Britain. Office for Budget Responsibility. Office for Budget Responsibility : Welfare trends report. 2019. 130 p.

13. Hall P. Impact Assessment (IA) Summary : Intervention and Options Summary : Analysis and Evidence Policy Option 1. Sustain Dev. 2011;1–28.

14. Department for Work and Pensions. Universal Credit: Further Information for Families. 2017; Available from: https://www.gov.uk/government/publications/universal-credit-and-your-family-quick-guide/universal-credit-further-information-for-families

15. Action F. Welfare that works better: 10 recommendations for improving the Universal Credit. London: Family Action; 2011.

16. Work and Pensions Committee. Universal Credit: the wait for a first payment [Internet].

2020. Available from: https://committees.parliament.uk/publications/3069/documents/28787/default/

17. Department for Work and Pensions. Universal Credit Statistical Ad Hoc : Length of Payment Delays for New Claims to Universal Credit. 2018.

18. Griffiths R, Wood M, Bennett F, Millar J. Uncharted Territory: Universal Credit, Couples and Money. 2020;

19. Britain Thinks. Learning from experiences of Universal Credit. London: Britain Thinks and Joseph Rowntree Foundation. 2018.
